# Joint Dynamic Trajectories of CEA and CA19-9 After Gastric Cancer Surgery: Prognostic Associations and a 12-Month Landmark Analysis

**DOI:** 10.3390/biomedicines14102175

**Published:** 2026-09-25

**Authors:** Yicong Zeng, Tong Deng, Jiali Du, Shiyu Tang, Yueyang Yu, Xudong Zhang, Lifa Li, Mengchi Jiang, Yumin Tang, Tong Zhou

**Affiliations:** 1 Second Department of Gastrointestinal Surgery, The Affiliated Hospital of North Sichuan Medical College, Nanchong 637000, China; a13550219507@163.com (Y.Z.); 18780720600@163.com (T.D.);; 2 National Clinical Key Specialty (General Surgery), Sichuan Clinical Research Center for Digestive Diseases, Sichuan Branch of National Clinical Research Center for Digestive Diseases, Nanchong 637000, China; 3 Division of Gastrointestinal Surgery, Department of General Surgery, West China Hospital of Sichuan University, Chengdu 610041, China

**Keywords:** machine learning, CA19-9, carcinoembryonic antigen, gastric cancer, survival analysis, trajectory analysis, landmark analysis

## Abstract

**Background**: Static single-time-point assessment of carcinoembryonic antigen and carbohydrate antigen 19-9 cannot capture postoperative temporal patterns. We evaluated whether their joint longitudinal trajectories were associated with survival after curative gastrectomy while explicitly addressing the time-dependent bias created when trajectory groups are defined using measurements obtained after surgery. **Methods**: We analyzed 208 patients with serial carcinoembryonic antigen and carbohydrate antigen 19-9 measurements. Five-time-point trajectories were retained as a descriptive and exploratory analysis. Primary prognostic inference used a 12-month landmark analysis restricted to patients alive and recurrence-free at 12 months with at least three observed paired measurements from the preoperative, 1-month, 6-month, and 12-month time points. Trajectories were re-derived using only pre-landmark information. Overall survival was defined by all-cause mortality. In leakage-controlled fivefold cross-validation, imputation, transformation, cluster selection, trajectory assignment, and model fitting were performed within each training fold. **Results**: The 12-month landmark cohort included 164 patients and yielded four joint trajectory groups: G1, *n* = 60; G2, *n* = 41; G3, *n* = 32; and G4, *n* = 31. After adjustment for age, sex, TNM stage, differentiation, tumor site, postoperative chemotherapy, and baseline log-transformed carcinoembryonic antigen and carbohydrate antigen 19-9, G4 was associated with higher point estimates for overall survival and disease-free survival but did not reach statistical significance, with hazard ratios of 1.81 and 1.90, respectively. Adding the landmark trajectory classification increased the apparent C-index from 0.689 to 0.710 for overall survival and from 0.711 to 0.723 for disease-free survival, while likelihood-ratio tests for incremental model fit were not significant. In leakage-controlled cross-validation, the Cox model with CTS4 achieved an out-of-fold C-index of 0.589 and a 24-month post-landmark AUC of 0.635, compared with 0.614 and 0.643 for logistic regression using the same inputs. The original five-time-point surgery-indexed analysis showed stronger associations but was retained only as exploratory because the exposure incorporated future follow-up information. **Conclusions**: Joint carcinoembryonic antigen and carbohydrate antigen 19-9 trajectories may contain prognostic information, but the association was materially attenuated after correction for exposure timing. These findings should therefore be considered exploratory and hypothesis-generating until prospective external validation is available.

## 1. Introduction

Gastric cancer ranks as the fifth most common malignancy worldwide [1]. Despite continuous refinements in surgical techniques and perioperative management, postoperative recurrence remains a major obstacle to long-term patient survival [2]. Serum tumor markers, owing to their simplicity and reproducibility, hold considerable value in postoperative surveillance for gastric cancer. However, the information obtained by the currently prevailing static single-time-point testing paradigm is limited to momentary measurements at individual time points. Consequently, the dynamic evolution of tumor burden during treatment and recovery cannot be captured. This constitutes a key bottleneck that constrains the prognostic predictive performance of these markers. There is therefore an urgent clinical need to develop a novel marker evaluation paradigm that integrates temporal information to optimize postoperative risk stratification in gastric cancer.

CEA is the most widely used serum biomarker for gastric cancer and has been incorporated into the recommendations of both the NCCN and Japanese gastric cancer treatment guidelines [3,4]. CA72-4 and CA19-9 have also been demonstrated to be associated with gastric cancer staging, recurrence, and prognosis [5]. Nevertheless, existing studies have largely focused on static levels of individual biomarkers at single time points, with a notable lack of systematic analysis addressing the combined dynamic changes of multiple markers [6]. This oversimplified approach has inherent limitations. On the one hand, a single marker is susceptible to interference from non-neoplastic conditions, resulting in suboptimal specificity. On the other hand, static thresholds fail to capture the temporal trends of marker evolution, potentially missing the early warning signals of recurrence or metastasis. From a clinical decision-making perspective, developing a method capable of integrating serial multi-marker information holds promise for breaking the current performance bottleneck of tumor marker application.

In recent years, trajectory analysis, a machine learning method, has gained increasing attention in oncology research. Its core strength lies in characterizing the temporal patterns of biomarker changes [7]. This approach has demonstrated superior prognostic discrimination over static indicators across multiple solid tumor types, including colorectal and lung cancers [8,9,10]. In the gastric cancer setting, several studies have explored postoperative trajectories of single markers such as CEA or CA72-4 [11,12]. However, the analysis of combined multi-marker trajectories remains an unexplored territory. Given that different markers may reflect distinct biological dimensions of tumors, with CEA associated with epithelial-derived tumor burden and CA19-9 with aberrant glycosylation, analyzing the trajectory of a single marker alone may fail to capture the synergistic prognostic information embedded in their joint evolution. This knowledge gap constitutes the direct impetus for the present study.

To address this gap, serial CEA and CA19-9 measurements were collected at five clinically relevant time points: preoperatively, 1 month postoperatively, 6 months postoperatively, 1 year postoperatively, and 2 or more years postoperatively. The full five-time-point trajectory was used to characterize longitudinal patterns. The preoperative baseline reflects initial tumor burden [13]. The early postoperative period reflects the effect of curative resection. The 6- to 12-month window captures dynamic changes following adjuvant therapy. The 2-year and beyond period covers the vast majority of recurrence events [14,15]. Because a trajectory defined using later follow-up measurements cannot be known at the date of surgery, a 12-month landmark analysis was added for prognostic inference. This analysis used only measurements available by the landmark and started the survival clock at 12 months among patients who were alive and recurrence-free at that time. We therefore aimed not only to describe a dual-marker Combined Trajectory Score, CTS4, but also to determine whether its prognostic signal persisted after explicit control of exposure timing and information leakage.

## 2. Methods

### 2.1. Study Design and Patient Inclusion/Exclusion Criteria

This was a single-center retrospective cohort study conducted at the Affiliated Hospital of North Sichuan Medical College. Eligible patients were 18 to 85 years of age, had histopathologically confirmed adenocarcinoma of the stomach or gastroesophageal junction, underwent R0 gastrectomy, had no distant metastasis at surgery, and had at least three originally observed paired CEA and CA19-9 measurements across the five planned time points. The screening exclusion categories were neoadjuvant treatment, preoperative distant or uncertain metastatic disease, a secondary malignancy or mixed histology, fewer than three originally observed paired tumor-marker time points, and loss of critical follow-up data. The requirement for at least three paired time points was applied before any interpolation or imputation and defined the longitudinal data completeness threshold used for trajectory eligibility.

Patient Selection Process:

From November 2019 to January 2023, 986 patients who underwent curative gastrectomy were screened. The mutually exclusive primary exclusion categories recorded in the screening log were neoadjuvant treatment in 182 patients, preoperative distant metastasis or uncertain metastatic status in 86, secondary malignancy or mixed histology in 35, fewer than three paired CEA and CA19-9 measurement time points in 369, and loss of critical follow-up data in 106. These exclusions accounted for all 778 patients not included in the trajectory cohort and left 208 patients for the original five-time-point trajectory analysis, as shown in Figure 1. Individual-level baseline data for all 778 excluded patients were not retained in the analytic export. A comparison with the 99 nonincluded patients for whom corresponding baseline extraction was available is therefore reported separately and is interpreted only as an available-data assessment rather than a complete screened-cohort comparison.

### 2.2. Postoperative Staging

Postoperative pathological classification was conducted based on the American Joint Committee on Cancer (AJCC) standards for all patients who received radical surgical treatment [16].

### 2.3. Tumor Marker Measurement and Trajectory Modeling

CEA and CA19-9 were measured using the Carcinoembryonic Antigen Reagent Kit (Roche Diagnostics GmbH, Mannheim, Germany) and the ARCHITECT CA19-9 XR Reagent Kit (Roche Diagnostics GmbH, Mannheim, Germany) with chemiluminescent microparticle immunoassay technology from the Institute of Hepatobiliary, Pancreatic, and Intestinal Diseases, North Sichuan Medical College.

Measurements were performed using a cobas e 801 automated immunoassay analyzer based on electrochemiluminescence immunoassay technology (Roche Diagnostics GmbH, Mannheim, Germany).

Serum carcinoembryonic antigen, CEA, was measured in ng/mL and carbohydrate antigen 19-9, CA19-9, was measured in U/mL at five planned time points: T1 preoperatively, T2 at approximately 1 month postoperatively, T3 at approximately 6 months, T4 at approximately 12 months, and T5 at 24 months or later. The exact marker date was used when available. For the landmark analysis, only T1 through T4 were eligible to contribute to trajectory construction.

For the original five-time-point analysis, raw values were truncated at 50 ng/mL for CEA and 370 U/mL for CA19-9 and then transformed using log(x + 1). Internal missing values with observed measurements immediately before and after the missing time point were filled by time-proportionally weighted linear interpolation. Endpoint missing values and isolated gaps were handled by multiple imputation with predictive mean matching using five imputations. The imputation model included age, sex, TNM stage, and the other available measurements of the same marker. Eligibility for trajectory analysis was based only on originally observed paired measurements. Imputed values were used only to complete the matrix required by the clustering algorithm.

Missingness was audited separately for each marker and time point in the 208-patient trajectory cohort. For CEA, missing values occurred in 0 patients at T1, 45 at T2, 24 at T3, 42 at T4, and 88 at T5. The corresponding counts for CA19-9 were 0, 45, 24, 41, and 88. Under the prespecified rule, 35 T2, 14 T3, and 13 T4 gaps were eligible for internal interpolation for each marker. The remaining missing values requiring imputation were 10 at T2, 10 at T3, 29 at T4, and 88 at T5 for CEA, and 10, 10, 28, and 88 for CA19-9. Overall, 60 patients required at least one interpolation and 98 required at least one imputed value. These data are summarized in Table S2.

A separate timing audit showed that the original full five-time-point trajectory construction did not censor biomarker measurements at the date of recurrence. Among the 87 patients with documented recurrence, 34 paired post-recurrence measurement occasions from 32 patients could therefore contribute to the descriptive five-time-point trajectory. To avoid interpreting established recurrence as a predictor of subsequent outcome, the five-time-point trajectory analyses were treated as exploratory in the revised manuscript. Primary prognostic inference was based on the 12-month landmark analysis described below, which included only patients alive and recurrence-free at the landmark and used only measurements obtained by that landmark.

For the descriptive five-time-point analysis, longitudinal K-means trajectory clustering was performed independently for CEA and CA19-9 using the R package kml, version 3.1. Candidate values of K from 2 to 5 were compared using the within-cluster sum of squares, Calinski–Harabasz index, silhouette coefficient, minimum cluster size, and resampling stability. K = 2 was retained for both markers. The resulting patterns were labeled low-stable and postoperative rebound. Agreement with standard K-means clustering was assessed using the adjusted Rand index.

### 2.4. Definition of Combined Trajectory Score (CTS4)

Based on whether CEA and CA19-9 each fell into the high-risk trajectory (postoperative rebound type), the 208 patients were classified into four combined trajectory subtypes (CTS4): Group 1 (both low-stable, *n* = 84); Group 2 (CA19-9 only rebound, *n* = 53); Group 3 (CEA only rebound, *n* = 36); and Group 4 (both postoperative rebound, *n* = 35). Group 1 (both low-stable) served as the reference group.

### 2.5. Twelve-Month Landmark Analysis and Leakage-Controlled Validation

To address immortal-time and time-dependent exposure bias, a 12-month landmark was selected before outcome modeling. Patients were eligible for the landmark cohort if they were alive and recurrence-free beyond 12 months and had at least three originally observed paired CEA and CA19-9 measurements among T1 through T4. Only pre-landmark marker information was used. Internal gaps were linearly interpolated using the planned observation times. Remaining missing pre-landmark values were imputed from age, sex, TNM stage, and the other pre-landmark marker values. Marker values were truncated at the same upper limits, transformed using log x plus 1, standardized, and clustered separately for CEA and CA19-9. Candidate K values from 2 to 5 were assessed using the Calinski–Harabasz index, silhouette coefficient, and minimum cluster size. The binary marker trajectories were then combined into landmark G1 through G4 groups. Overall survival and disease-free survival follow-up began at the 12-month landmark.

For leakage-controlled fivefold cross-validation, every data-dependent operation was repeated within each outer training fold. This included interpolation, multivariable imputation, transformation, scaling, selection of the number of clusters, fitting of marker-specific clusters, assignment of CTS4, and predictive model fitting. The learned preprocessing and cluster centroids were then applied to the held-out fold without using its outcomes. To reduce instability in the modest landmark cohort, model hyperparameters were prespecified rather than selected on the complete dataset. Performance was summarized using out-of-fold Harrell C-index and the AUC for death within 24 months after the landmark, corresponding to 36 months after surgery.

### 2.6. Statistical Analysis

Continuous variables were summarized as medians with interquartile ranges and categorical variables as counts and percentages. Between-group comparisons used the Kruskal–Wallis test, chi-square test, or Fisher exact test as appropriate. Overall survival was defined as time to death from any cause. Disease-free survival was defined as time to first documented recurrence or death from any cause. In the primary landmark analysis, time zero was the 12-month landmark. Cox models adjusted for age, sex, TNM stage, differentiation, tumor site, postoperative chemotherapy, baseline log-transformed CEA, and baseline log-transformed CA19-9. Incremental model fit after adding CTS4 was evaluated using likelihood-ratio tests and changes in the Harrell C-index. The original five-time-point analyses indexed from surgery, including Kaplan–Meier, LASSO–Cox, standard Cox, subgroup, and machine-learning analyses, were retained as exploratory analyses for comparison with the original submission. Proportional hazards assumptions were assessed with Schoenfeld residuals. Statistical tests were two-sided with alpha = 0.05.

Software: Statistical analyses were performed using R software (version 4.3.1; R Foundation for Statistical Computing, Vienna, Austria).

## 3. Results

### 3.1. Patient Characteristics

Of the 986 screened patients, 208 met the requirements for the original five-time-point trajectory cohort. Their median age was 65.0 years with an interquartile range of 57.0 to 71.0 years, and 155 patients were male. Stage I, II, and III disease was present in 59, 45, and 104 patients, respectively. Poorly differentiated, undifferentiated, or special histologic types were present in 129 patients, and 167 received postoperative chemotherapy. Baseline characteristics by the original CTS4 group are shown in Table 1. Age, sex, and both baseline tumor-marker concentrations differed across the original CTS4 groups, whereas TNM stage and tumor site did not. A baseline comparison was possible for 99 nonincluded patients retained in the analytic extraction. No statistically significant differences were identified for age, sex, TNM stage, differentiation, tumor site, or postoperative chemotherapy, although the nonincluded subset had fewer paired marker observations, as expected from the selection mechanism. Because survival data were available for only 20 out of these 99 patients, survival was not compared. These available-data comparisons are shown in Table S1. Admission year could be recovered for 203 out of the 208 analyzed patients from the deidentified source archive: 3 were admitted in 2019, 19 in 2020, 78 in 2021, 102 in 2022, and 1 in 2023. Five admission dates were not retained in the deidentified extraction. The administrative censoring date was confirmed as 30 June 2026. Recalculation with the reverse Kaplan–Meier method confirmed a median follow-up of 58 months with a 95% confidence interval of 53 to 60 months. The reverse Kaplan–Meier estimator treats deaths as censored and estimates the distribution of potential follow-up from censoring times; therefore, its median can exceed the simple median calendar time from enrollment when earlier deaths are common.

### 3.2. Trajectory Clustering of CEA and CA19-9

Given the presence of outliers in the CEA and CA19-9 data, a natural logarithmic transformation [log(test value + 1)] was applied to normalize these values and approximate a normal distribution. Subsequently, the longitudinal data for CEA and CA19-9 from the preoperative period to beyond 2 years postoperatively in the 208 patients were separately subjected to KML trajectory clustering using the kml package in R (version 4.3.1) to classify their trajectories [7]. The kml approach extends the k-means clustering framework for longitudinal data and identifies latent subgroups according to individual temporal patterns without requiring predefined assumptions regarding trajectory distributions [17].

Candidate models with K = 2–5 clusters were evaluated by integrating the Calinski–Harabasz (CH) index, average silhouette coefficient, smallest cluster size, and resampling consistency. The CH index reflects the separation between clusters, whereas the silhouette coefficient evaluates both within-cluster cohesion and between-cluster separation [18]. Resampling consistency was used to assess the robustness of clustering results under repeated sample perturbation [19]. Based on these evaluation criteria, both the CEA and CA19-9 trajectories demonstrated the best clustering performance with K = 2.

For the CEA trajectories, the K = 2 model showed a CH index of 148.9, a silhouette coefficient of 0.403, a smallest cluster size of 71 patients, and mean/minimum resampling consistency values of 0.901/0.874. In comparison, the K = 3 model resulted in lower values for these parameters (CH index, 125.5; silhouette coefficient, 0.390; smallest cluster size, 5 patients; resampling consistency, 0.823/0.670). Moreover, K = 3 generated a small cluster containing only five patients, indicating that further increasing the number of clusters reduced cluster balance and compromised classification stability. For CA19-9, the two-cluster solution yielded a Calinski–Harabasz index of 157.3, a silhouette score of 0.370, mean/minimum resampling consistency of 0.924/0.915, and a smallest cluster comprising 88 patients. When three clusters were specified, the corresponding values were 136.0, 0.339, 0.866/0.766, and 21 patients. Increasing K further to 4 or 5 led to lower CH indices and silhouette scores, with clusters becoming smaller and less reproducible. Based on these comparisons, K = 2 was retained as the primary specification for both the CEA and CA19-9 trajectories.

With the two-cluster model, CEA trajectories fell into a low-stable group (*n* = 137) and a postoperative rebound group (*n* = 71). The low-stable group maintained low CEA levels across the preoperative and postoperative follow-up, with only a slight rise during the late observation period. In contrast, the postoperative rebound group had higher baseline CEA concentrations, an early postoperative decline, and a subsequent increase after 6–12 months that persisted over later follow-up. CA19-9 trajectories exhibited an analogous two-group pattern: 120 patients followed a low-stable trajectory, and 88 patients followed a postoperative rebound trajectory. Despite some within-group variation, the mean trajectories remained clearly separated, supporting the two-cluster classification.

As a sensitivity analysis, standard k-means clustering was also performed to check the robustness of these trajectory assignments. Agreement between the two clustering approaches was evaluated using the adjusted Rand index (ARI) and maximum label matching rate [20]. Under the K = 2 model, the ARI between kml and k-means clustering for CEA was 0.887, with a maximum matching rate of 97.1%. For CA19-9, the two algorithms generated identical classifications (ARI = 1.000, matching rate = 100%). Under the K = 3 model, CEA maintained complete agreement between algorithms (ARI = 1.000), whereas CA19-9 showed slightly reduced consistency (ARI = 0.905, matching rate = 96.6%). These findings further supported the stability of the K = 2 trajectory model.

The binary trajectories of CEA and CA19-9 were subsequently combined to generate four combined trajectory subtypes (CTS). CTS4G1 (both low-stable subtype) included 84 patients (40.4%), CTS4G2 (CA19-9-only postoperative-rebound subtype) included 53 patients (25.5%), CTS4G3 (CEA-only postoperative-rebound subtype) included 36 patients (17.3%), and CTS4G4 (dual-postoperative-rebound subtype) included 35 patients (16.8%). These CTS categories corresponded closely with the individual marker trajectories. Specifically, CTS4G1 and CTS4G2 together comprised 137 patients, corresponding to the CEA low-stable trajectory; CTS4G3 and CTS4G4 together comprised 71 patients, corresponding to the CEA postoperative rebound trajectory. Similarly, CTS4G1 and CTS4G3 together included 120 patients, corresponding to the CA19-9 low-stable trajectory, whereas CTS4G2 and CTS4G4 included 88 patients, corresponding to the CA19-9 postoperative rebound trajectory. The combined trajectory-based classification captured the longitudinal patterns of both tumor markers while preserving the complementary dynamic information each marker provides.

Across the original 208-patient cohort, CEA and CA19-9 each followed two principal longitudinal patterns, a low-stable trajectory and a postoperative rebound trajectory. Combining the two marker classifications yielded four descriptive CTS4 subtypes. The two-cluster solution was retained because it provided a favorable balance of separation, minimum cluster size, and resampling stability. The full five-time-point distributions and trajectories are shown in Figure 2. Because these trajectories use measurements accrued after surgery and can include post-recurrence measurements, subsequent surgery-indexed survival analyses of this original classification are reported only as exploratory analyses.

### 3.3. Twelve-Month Landmark Analysis

The landmark cohort included 164 patients who were alive and recurrence-free beyond 12 months and had at least three observed paired measurements among T1 through T4. Ninety-three patients had all four paired time points and 71 had three. In the landmark data, K = 2 was retained for both markers. The Calinski–Harabasz index was 119.33 for CEA and 133.75 for CA19-9, with minimum cluster sizes of 63 and 72 patients, respectively. The resulting landmark CTS4 distribution was G1 *n* = 60, G2 *n* = 41, G3 *n* = 32, and G4 *n* = 31. There were 62 deaths and 68 disease-free survival events after the landmark.

In the fully adjusted landmark Cox model, the hazard ratio for G4 versus G1 was 1.81 for overall survival, with a 95% confidence interval of 0.67 to 4.89 and *p* = 0.242, and 1.90 for disease-free survival, with a 95% confidence interval of 0.74 to 4.86 and *p* = 0.183. G2 and G3 were also not statistically significant. Adding landmark CTS4 to the clinical and baseline-marker model increased the apparent C-index from 0.689 to 0.710 for overall survival and from 0.711 to 0.723 for disease-free survival. The corresponding 24-month post-landmark AUC values increased from 0.736 to 0.760 and from 0.755 to 0.769. However, likelihood-ratio tests for the three added CTS4 terms were not significant for overall survival or disease-free survival, with *p* = 0.583 and *p* = 0.569. The landmark effect estimates and model comparisons are summarized in Table S3.

### 3.4. Exploratory Full-Trajectory Surgery-Indexed Survival Analysis

For transparency and comparability with the original submission, the original five-time-point CTS4 classification was also analyzed with time indexed from the date of surgery. These analyses are exploratory because CTS4 was not knowable at surgery. Overall survival was defined as time from surgery to death from any cause. Disease-free survival was defined as time to first recurrence or death from any cause. Living patients without a disease-free survival event were censored at the last documented contact or at the administrative censoring date of 30 June 2026, whichever occurred first. The reverse Kaplan–Meier estimate of median follow-up was 58 months. In these exploratory surgery-indexed analyses, the four CTS4 groups showed significant Kaplan–Meier separation for both outcomes, as shown in Figure 3. These findings are not interpreted as bias-corrected prognostic estimates and are secondary to the landmark analysis.

Univariate Cox proportional hazards regression in the exploratory surgery-indexed analysis showed the strongest adverse signal in G4. Compared with G1, G4 was associated with an overall survival hazard ratio of 2.58, with a 95% confidence interval of 1.51 to 4.41 and *p* = 0.001, and a disease-free survival hazard ratio of 2.66, with a 95% confidence interval of 1.59 to 4.46 and *p* < 0.001. Within the four-group CTS4 model, G2 and G3 did not reach statistical significance. Specifically, G2 versus G1 had a hazard ratio of 1.34 for overall survival, with a 95% confidence interval of 0.79 to 2.27 and *p* = 0.271, and 1.30 for disease-free survival, with a 95% confidence interval of 0.78 to 2.18 and *p* = 0.310. G3 versus G1 had a hazard ratio of 1.44 for overall survival, with a 95% confidence interval of 0.81 to 2.56 and *p* = 0.210, and 1.49 for disease-free survival, with a 95% confidence interval of 0.85 to 2.61 and *p* = 0.162. These nonsignificant subgroup contrasts are not interpreted as evidence that isolated single-marker trajectories lack prognostic information, because the separate CA19-9 trajectory analysis showed a significant association and G2 and G3 had limited sample sizes.

Beyond CTS4 classification, conventional tumor-related factors were also associated with survival outcomes in the exploratory univariable analyses. Higher TNM stage and nodal stage were associated with worse outcomes, while postoperative chemotherapy showed a protective association. The complete univariable estimates are presented in Table 2. These surgery-indexed results should be interpreted within the timing limitations described above.

### 3.5. Exploratory LASSO–Cox Analysis of the Full Five-Time-Point CTS4

For comparison with the original submission, LASSO-penalized Cox regression was retained as an exploratory full five-time-point analysis. The penalty parameter was selected by 10-fold cross-validation. The resulting coefficient paths and cross-validation curves are shown in Figure 4A–D. Refitted hazard-ratio estimates are summarized in Table 3 and Figure 4E. Because the CTS4 exposure in this analysis was derived using follow-up measurements obtained after surgery, these estimates were not used as the revised primary prognostic evidence.

In the exploratory full-trajectory LASSO–Cox analysis, G4 remained associated with higher risk than G1 after adjustment for age and TNM stage, with a hazard ratio of 2.25 for overall survival, a 95% confidence interval of 1.26 to 4.04, and *p* = 0.006, and a hazard ratio of 2.29 for disease-free survival, a 95% confidence interval of 1.30 to 4.02, and *p* = 0.004. The G2 and G3 contrasts were not statistically significant. G2 versus G1 had a hazard ratio of 1.36 for overall survival, a 95% confidence interval of 0.80 to 2.32, and *p* = 0.257, and a hazard ratio of 1.33 for disease-free survival, a 95% confidence interval of 0.79 to 2.24, and *p* = 0.279. G3 versus G1 had a hazard ratio of 1.18 for overall survival, a 95% confidence interval of 0.65 to 2.14, and *p* = 0.594, and a hazard ratio of 1.21 for disease-free survival, a 95% confidence interval of 0.67 to 2.18, and *p* = 0.522. Because the exposure was derived from the full five-time-point trajectory, these results are retained only as exploratory evidence.

Age was not independently associated with overall survival or disease-free survival in either exploratory LASSO–Cox model. The hazard ratio for age was 1.01 in the overall survival model, with a 95% confidence interval of 0.99 to 1.04 and *p* = 0.249, and 1.01 in the disease-free survival model, with a 95% confidence interval of 0.99 to 1.04 and *p* = 0.235. TNM stage showed a stepwise association with risk. Using stage I as the reference, stage II had a hazard ratio of 2.67 for overall survival and 2.85 for disease-free survival, while stage III had corresponding hazard ratios of 6.31 and 7.26. Thus, advanced TNM stage was consistently associated with outcome in this exploratory model, while the CTS4 coefficient should be interpreted in light of the exposure-timing limitation.

Based on predictors retained in the exploratory OS model, a nomogram was constructed for descriptive purposes and is shown in Figure 4F. This nomogram is not proposed for clinical deployment because the underlying full-trajectory exposure is subject to time-dependent bias and has not been externally validated.

### 3.6. Exploratory Standard Multivariable Cox Regression

As an exploratory complement to the LASSO–Cox analysis of the original five-time-point exposure, conventional multivariable Cox regression was performed using the prespecified clinical covariates. Separate models were fitted for the CEA trajectory, CA19-9 trajectory, and joint CTS4 classification. Each exploratory model adjusted for age, sex, TNM stage, histological differentiation, tumor location, and postoperative chemotherapy.

In the exploratory single-marker standard Cox models, the CEA postoperative rebound trajectory was not statistically significant, whereas the CA19-9 postoperative rebound trajectory was associated with worse overall survival and disease-free survival, with hazard ratios of 1.68 and 1.58 and *p* values of 0.019 and 0.033, respectively. These results show that the lack of significance for the isolated G2 and G3 categories in the four-group CTS4 model should not be interpreted as evidence that a single-marker trajectory carries no prognostic information. The detailed results of the standard multivariable Cox regression analyses of CEA trajectory and CA19-9 trajectory in relation to survival outcomes are presented in Table 4 and Table 5, respectively.

In the original surgery-indexed CTS4 model, G4 remained associated with worse outcomes after adjustment for conventional clinicopathological covariates. Because the CTS4 groups also differed in baseline CEA and CA19-9 concentrations, an additional model was fitted with both baseline markers entered as continuous log-transformed covariates. In that model, the G4 versus G1 hazard ratio was 2.46 for overall survival, with a 95% confidence interval of 1.07 to 5.68 and *p* = 0.035, and 2.71 for disease-free survival, with a 95% confidence interval of 1.21 to 6.06 and *p* = 0.016. Nevertheless, the incremental contribution of CTS4 beyond the clinical plus baseline-marker model was modest. The C-index changed from 0.729 to 0.741 for overall survival and from 0.737 to 0.750 for disease-free survival, while likelihood-ratio *p* values were 0.221 and 0.116. These estimates remain exploratory because of exposure timing. The detailed results of the standard multivariable Cox regression analysis of combined CEA/CA19-9 trajectory subtypes (CTS4) and survival outcomes are presented in Table 6.

### 3.7. Exploratory Apparent Predictive Performance

Apparent full-cohort discrimination of the exploratory surgery-indexed models is summarized in Table 7 and Figure 5. In the original clinical model, the C-index was 0.718 for overall survival and 0.727 for disease-free survival. Adding the full five-time-point CTS4 increased these values to 0.739 and 0.750, respectively. The previously truncated disease-free survival value in the Abstract has therefore been corrected in the revised manuscript. Because these estimates are apparent performance from the complete dataset and the full CTS4 exposure uses future measurements, they are not compared directly with out-of-fold machine-learning estimates and are not used as the primary performance claim.

### 3.8. Leakage-Controlled Machine Learning Comparison

A leakage-controlled fivefold comparison was performed in the 12-month landmark cohort. All preprocessing and trajectory derivations were repeated within the training portion of each fold. The Cox clinical plus baseline-marker model achieved an out-of-fold C-index of 0.574 and a 24-month post-landmark AUC of 0.601. Adding CTS4 increased these values to 0.589 and 0.635. Among the eight machine-learning models using the same clinical, baseline-marker, and fold-specific CTS4 inputs, XGBoost had the highest AUC at 0.648, followed by logistic regression at 0.643 and elastic net at 0.642. Random forest had the highest C-index among the machine-learning classifiers at 0.629. Differences were small, and overall performance was modest. The corrected out-of-fold comparison is presented in Table 8. The visual diagnostics in Figure 6, Figure 7, Figure 8, Figure 9 and Figure 10 are retained as exploratory displays of the original full-trajectory modeling workflow and are not the basis for the revised primary performance conclusions.

### 3.9. Exploratory Subgroup Analysis

The exploratory full-trajectory comparison between G4 and G1 across clinical strata is summarized in Table 9 and Figure 11. These subgroup estimates are presented for descriptive completeness only. The modest stratum-specific event counts and the time-dependent nature of the original CTS4 exposure preclude firm claims of effect modification.

### 3.10. Sensitivity Analyses

The multiple-imputation sensitivity analysis of the original surgery-indexed CTS4 exposure produced estimates similar to the corresponding complete-data exploratory analysis, as shown in Table 10. This finding indicates that the original association was not driven primarily by the selected missing-data procedure, but it does not resolve the exposure-timing bias addressed by the landmark analysis.

When the original CTS4 exposure was collapsed into three descriptive risk groups, the highest-risk group remained associated with worse overall survival in the surgery-indexed exploratory model. This analysis is presented only as a robustness check of the original descriptive classification and is not treated as bias-corrected prognostic evidence.

Changing the original descriptive clustering specification from K = 2 to K = 3 produced a similar apparent overall survival C-index, 0.596 versus 0.597. Together with the cluster-balance and stability diagnostics, this supported retaining K = 2 for descriptive trajectory characterization. This sensitivity analysis does not address the temporal definition of the exposure.

## 4. Discussion

This study evaluated joint longitudinal patterns of CEA and CA19-9 after curative gastrectomy, and after review, explicitly separated descriptive full-trajectory associations from bias-controlled prognostic inference. The original five-time-point CTS4 groups showed clear surgery-indexed survival separation. However, these groups were defined using measurements obtained after the survival clock had started, and some late measurements occurred after recurrence. When trajectories were re-derived using only information available by a 12-month landmark and follow-up began at that landmark, the G4 effect estimate remained in the adverse direction but was smaller and no longer statistically significant. The revised interpretation is therefore more conservative: joint trajectories may contain prognostic information, but this dataset does not establish CTS4 as a robust independent predictor once exposure timing is handled explicitly.

CEA and CA19-9 are the most commonly used serum tumor markers in the diagnosis and management of gastric cancer. The reference threshold for the former is typically 5 ng/mL, whereas that for the latter is 37 U/mL. A systematic review conducted by the Japanese Gastric Cancer Association Working Group included 187 studies on CEA and CA19-9, reporting positivity rates of 21.1% and 27.8%, respectively, and demonstrating that both markers were significantly correlated with tumor stage and patient survival. Although serum markers are of limited diagnostic value for early-stage cancer and lack satisfactory specificity, they play a well-established role in recurrence detection, the prediction of distant metastasis, survival prognostication, and postoperative surveillance. A positive conversion of these markers typically precedes radiological abnormalities by 2 to 3 months [5].

The prognostic superiority of dynamic trajectory analysis over static single-time-point measurements merits discussion. Conventional approaches rely on fixed cutoff values applied to solitary measurements. This practice assumes that marker levels remain stable over time, which is seldom the case in clinical reality. Postoperative marker levels are influenced by multiple factors, including residual tumor burden, host immune response, and the effects of adjuvant therapy. A single measurement may be falsely reassuring if taken during a transient decline, or falsely alarming if affected by benign fluctuations. Trajectory analysis, in contrast, integrates information across multiple time points and thereby captures the underlying trend of tumor evolution. This methodological advantage explains why trajectory-based classification demonstrated stronger prognostic discrimination in the present cohort.

Numerous previous studies have confirmed that the expression of CEA, CA72-4, and CA19-9 in gastric cancer is closely associated with tumor stage, chemotherapy response, and recurrence. However, single markers are constrained by limitations in both sensitivity and specificity. Combined detection has been demonstrated to improve diagnostic efficacy [21,22]. Luan et al. analyzed 368 patients with stages I–III gastric cancer who underwent radical resection and found that preoperative positivity for CA19-9 and CA242 were independent risk factors for recurrence or metastasis, with postoperative CA19-9 yielding the highest AUC value [14]. Additional evidence indicates that postoperative CA19-9 is an independent prognostic factor for recurrence-free survival and overall survival in patients with stage III gastric cancer [23]. Regarding marker combinations, Zhang et al., based on data from 1966 gastric cancer patients, reported that the combined number of elevated preoperative and postoperative CEA, CA72-4, and CA19-9 (stratified as C0–C3 groups) exhibited a significant stepwise decrease in 5-year overall survival, with the combined marker C-index (0.65–0.66) substantially outperforming that of single markers (0.53–0.56) [24]. Nevertheless, these studies predominantly focused on static cutoff values at specific time points, which inadequately capture the dynamic evolution of markers during treatment. In recent years, longitudinal trajectory analysis has demonstrated superior prognostic discrimination over single-point assessments across multiple solid tumor types. In the gastric cancer setting, a machine learning study based on 578 patients employed k-means clustering to identify three perioperative CEA trajectories (high, intermediate, and low), revealing that higher CEA trajectories were significantly associated with worse disease-free survival and overall survival. The high-trajectory group exhibited a 2.64-fold higher mortality risk compared to the low-trajectory group [6]. Furthermore, CEA trajectories have also been applied to predict pathological complete response following neoadjuvant chemotherapy in locally advanced gastric cancer [11]. In the context of CA19-9, studies have explored its dynamic changes during neoadjuvant chemotherapy for triple-negative gastric cancer, identifying CA72-4 trajectory grouping as an independent prognostic factor [12]. However, combined trajectory analysis of perioperative CEA and CA19-9 dual biomarkers remains unexplored in gastric cancer patients.

To our knowledge, this study is among the first to examine joint perioperative CEA and CA19-9 trajectories in gastric cancer. The revised analysis separates descriptive full-trajectory patterns from a landmark-based prognostic assessment, thereby avoiding a claim of validation that is not supported by this single-center retrospective cohort.

The single-marker and four-group results also require careful reconciliation. In the exploratory whole-cohort model, the CA19-9 postoperative rebound trajectory was significantly associated with overall survival and disease-free survival, whereas the isolated G2 and G3 categories in the four-group CTS4 model were not statistically significant. These are not contradictory findings. Partitioning the cohort into G2 and G3 reduced the available sample sizes to 53 and 36 patients in the original classification and further widened the confidence intervals. Approximate detectable-effect calculations showed that for 80% power at a two-sided alpha of 0.05, the G2 versus G1 comparison would require hazard ratios of about 2.14 for overall survival and 2.10 for disease-free survival, while the G3 versus G1 comparison would require about 2.35 and 2.30. Moderate single-marker effects could therefore remain undetected in those smaller contrasts. The non-significant G2 and G3 coefficients should be interpreted as imprecise evidence rather than evidence of no signal, and the previous biological narrative that dual-marker encoding filters out single-marker noise has been removed.

The k-means framework offers computational efficiency and interpretability. In this study, cluster number was assessed using the Calinski–Harabasz index, silhouette coefficient, minimum cluster size, resampling stability, and agreement with standard k-means by adjusted Rand index. These diagnostics supported K = 2 as the most stable and clinically interpretable partition in this dataset.

The adverse direction of the double-rebound pattern remains biologically plausible. Persistent or recurrent elevation of both markers can accompany residual or recurrent disease and may reflect a greater tumor burden or aggressive biology [25,26]. At the same time, the timing audit in this study shows why this explanation cannot be used as proof of prospective predictive value. Some late marker measurements in the original trajectory were obtained after recurrence, and the baseline CEA and CA19-9 levels were already higher in G4. Thus, part of the full-trajectory association may reflect pre-existing disease severity or established progression. The baseline-marker-adjusted exploratory model retained a G4 association, but the incremental likelihood-ratio tests were not significant and the landmark analysis was attenuated. These observations argue for prospective trajectory definitions based on measurements accrued before the prediction origin.

From a clinical standpoint, serial CEA and CA19-9 measurements remain attractive because they are inexpensive and routinely available. The present results support further evaluation of joint longitudinal monitoring, but they do not justify changing surveillance intervals or triggering imaging solely on the basis of CTS4. Any future clinical rule should define a fixed prediction origin, exclude post-event biomarker information, and be validated prospectively in an independent cohort before it is used to intensify or de-escalate follow-up.

Several limitations should be emphasized. First, the retrospective single-center design created substantial selection through the requirement for serial tumor-marker measurements. Although the screening log accounted for all 778 exclusions, individual-level clinicopathological data were retained for only 99 nonincluded patients in the analytic extraction, so selection bias cannot be fully quantified. Second, the original five-time-point CTS4 exposure incorporated information accrued after surgery, and in 32 recurrent patients, included at least one post-recurrence measurement occasion. Those analyses are therefore exploratory and potentially affected by immortal-time and reverse-temporality bias. The 12-month landmark analysis was added to address this concern, but it necessarily changes the target population to patients alive and recurrence-free at 12 months and reduces the sample size. Third, missing marker values were common at later time points, particularly T5, and required interpolation or imputation. Fourth, subgroup comparisons had limited power, especially for G2 and G3. After multivariable adjustment, modest associations may have been attenuated, which may partly explain the less pronounced prognostic effects observed for some individual-marker trajectories. The combined CEA/CA19-9 trajectory classification, however, integrates complementary longitudinal information from both biomarkers and may therefore provide a more comprehensive characterization of postoperative disease dynamics than either marker alone. This finding supports the rationale for dual-marker integration and is broadly consistent with previous work by Kochi et al. [27]. Fifth, no external validation cohort was available. The leakage-controlled cross-validation reduces internal optimism but cannot replace external prospective validation.

The study also has methodological strengths. The k-means clustering algorithm employed in this study offers advantages of high computational efficiency and strong interpretability [7,28]. Serial measurements of two routinely used markers were jointly analyzed, clustering stability was examined using multiple internal criteria, missingness was quantified transparently, and the revised analysis directly addresses the timing of exposure definition. In addition, preprocessing and trajectory derivation were repeated within each cross-validation training fold in the corrected validation analysis. These steps improve dthe transparency of the evidence while also showing that the more conservative, bias-controlled estimates were weaker than the original surgery-indexed associations.

Taken together, the original five-time-point CTS4 classification captured clinically recognizable longitudinal patterns and showed exploratory associations with outcome. However, the 12-month landmark analysis attenuated the association and did not demonstrate a statistically significant independent effect of the G4 trajectory. The findings therefore support prospective evaluation of joint CEA and CA19-9 trajectories rather than immediate clinical adoption of CTS4.

## 5. Conclusions

In conclusion, joint CEA and CA19-9 trajectories after gastrectomy may provide complementary information beyond isolated measurements, but their prognostic value is sensitive to how the prediction origin is defined. In this cohort, the adverse G4 pattern observed in the original full-trajectory analysis was attenuated and not statistically significant in a 12-month landmark analysis. CTS4 should therefore be regarded as an exploratory framework that requires prospective, multicenter external validation before it can guide postoperative surveillance.

## Figures and Tables

**Figure 1:**
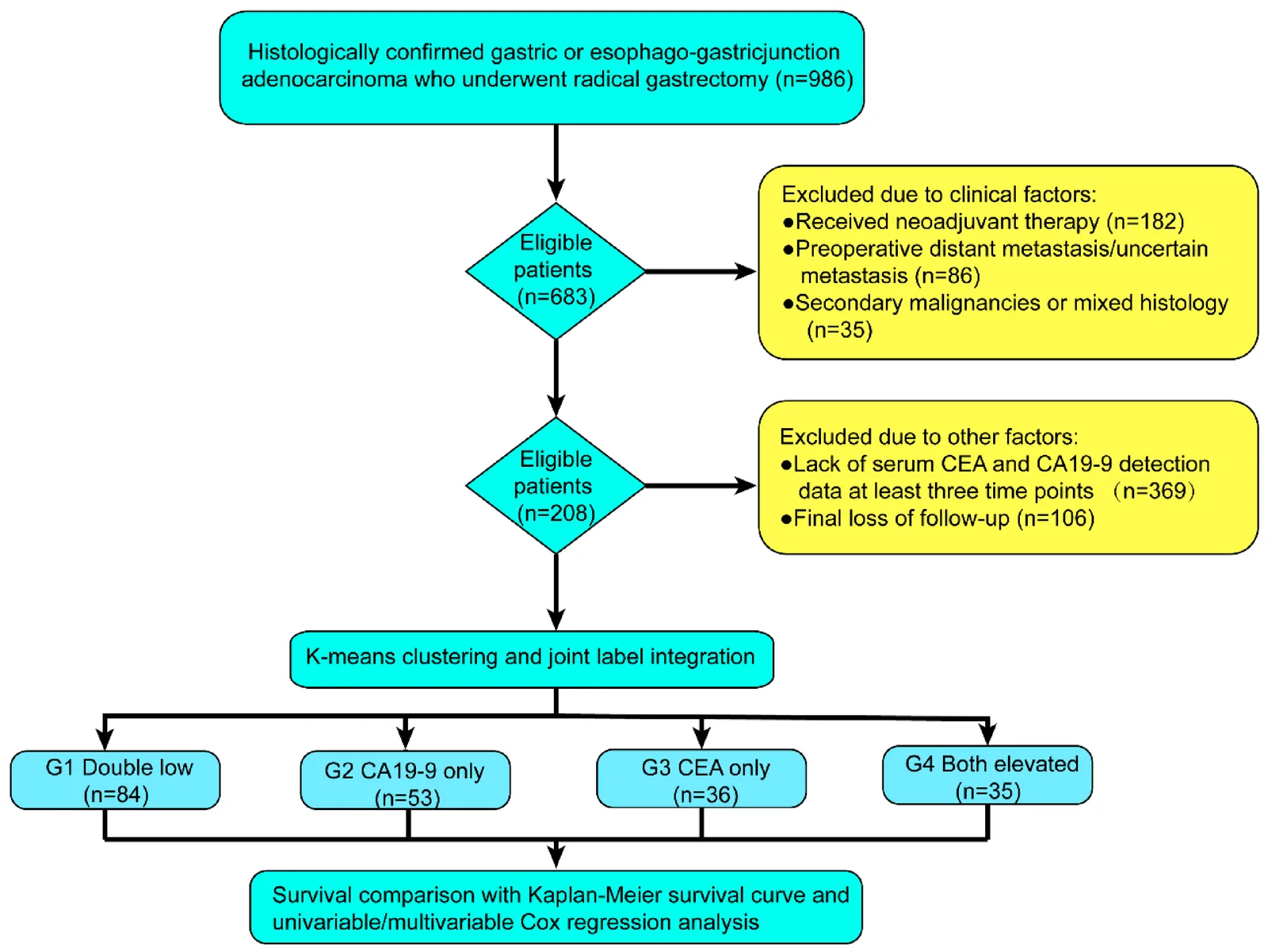
Study flowchart showing patient selection and the original descriptive five-time-point CEA and CA19-9 trajectory classification. The prognostic reanalysis in the revised manuscript uses a separate 12-month landmark cohort described in Section 2.5.

**Figure 2:**
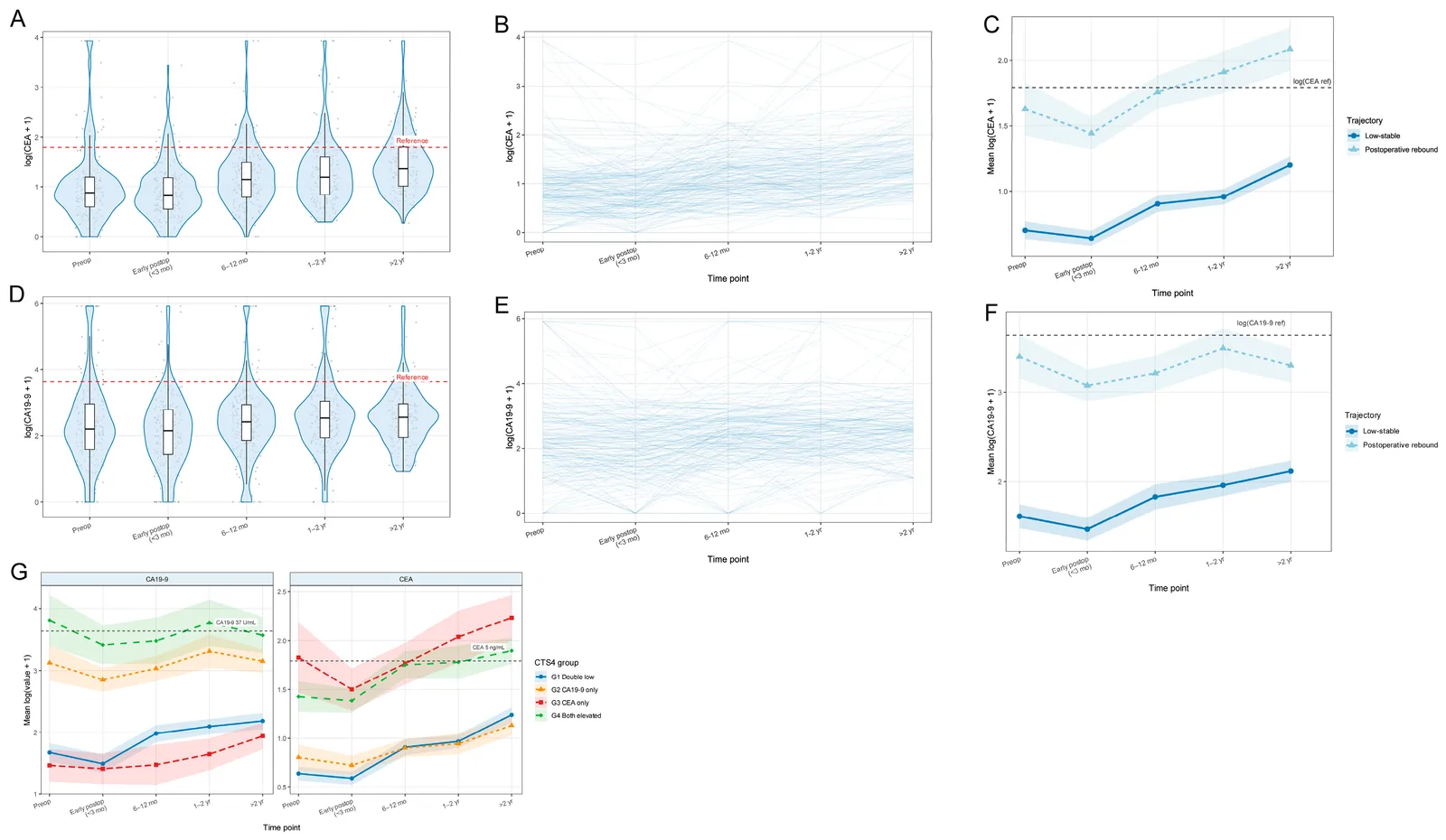
(**A**,**D**) Distributions of log(x + 1)-transformed CEA and CA19-9 values across the five follow-up time points. Violin plots depict the density contours of the data, with individual observations overlaid as dots; the red dashed line marks the reference level. Gray dots represent the raw observations for individual patients at the corresponding time points. The y-axis represents log(CEA + 1) or log(CA19-9 + 1), as appropriate. (**B**,**E**) Individual trajectory plots for CEA and CA19-9, respectively. Each thin line traces the log(CEA + 1) or log(CA19-9 + 1) course of a single patient over time, and the overall pattern shows an early postoperative decline that is frequently followed by a rebound. (**C**,**F**) Trajectory clusters identified by KML for CEA and CA19-9. Solid lines represent the group-level mean log(CEA + 1) and log(CA19-9 + 1) values at the five time points, with shaded bands indicating the uncertainty ranges of these means; these panels highlight the dynamic separation between the low-stable trajectory and the higher or rebound trajectory for each marker. (**G**) Combined dynamic trajectories of CEA and CA19-9 across the four CTS subtypes. This panel simultaneously plots the mean changes of both biomarkers in CTS4 G1, G2, G3, and G4, making it clear that the high-risk CTS4 G4 group maintains persistently higher levels or a rebound trend in both biomarker dimensions.

**Figure 3:**
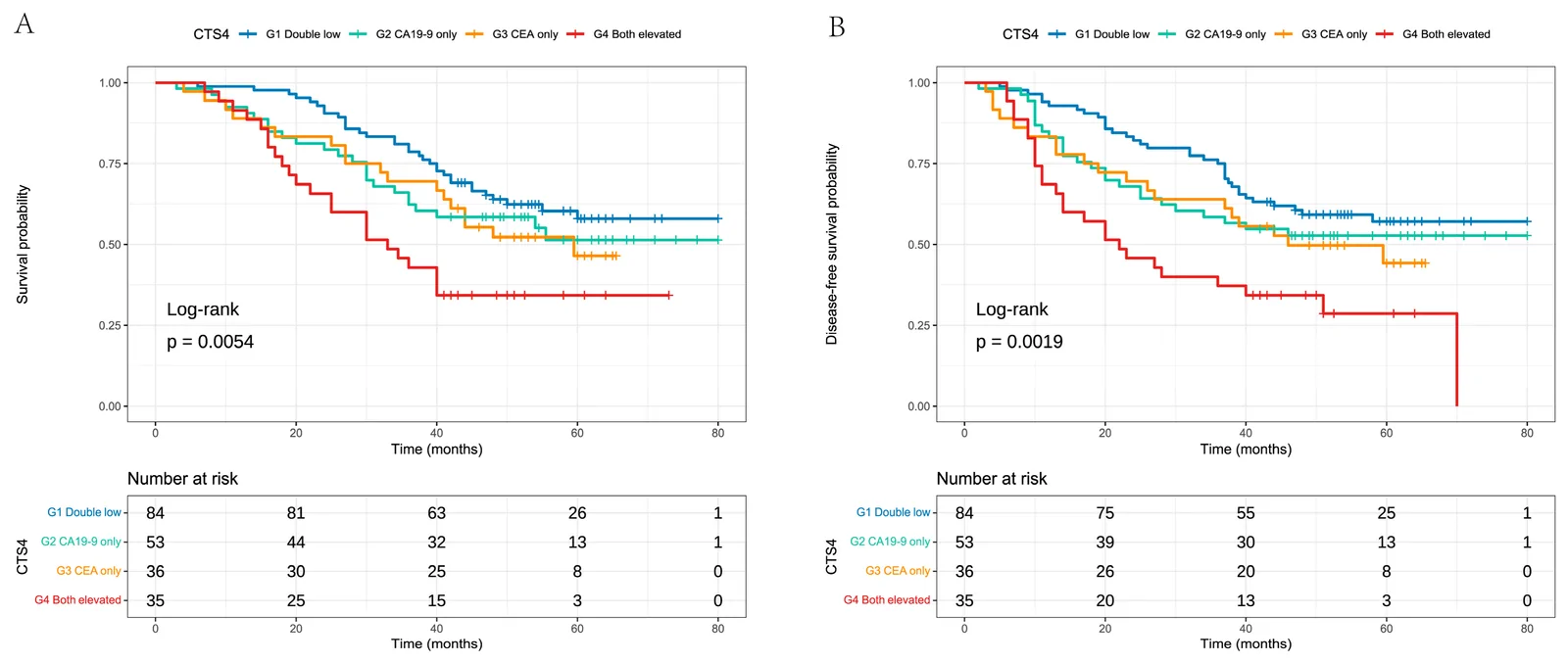
(**A**) Kaplan–Meier OS curves by CTS subgroup. The curves show changes in survival probability over the follow-up period for different CTS subtypes; the risk table below shows the number of patients still at risk at each time point. This figure is used to assess the unadjusted association between CTS subtypes and overall survival (OS) outcomes. (**B**) Kaplan–Meier DFS curves by CTS subgroup. DFS events include recurrence or death; this figure is used to assess the unadjusted association between CTS subtypes and the composite outcome of recurrence or death (disease-free survival [DFS]). These surgery-indexed curves use the original five-time-point CTS4 classification and are presented as exploratory analyses because group assignment depends on follow-up measurements obtained after surgery.

**Figure 4:**
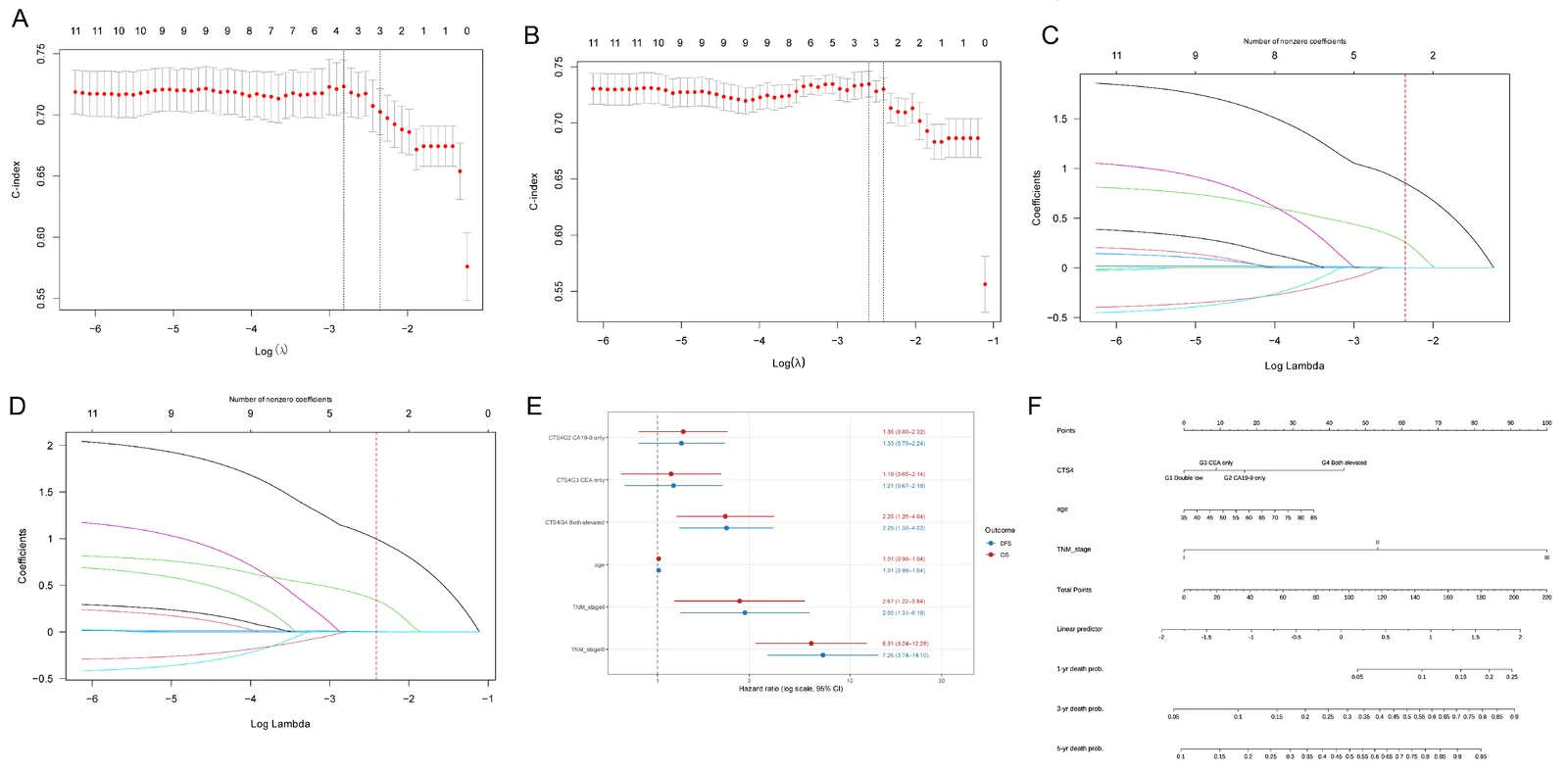
(**A**,**B**) Cross-validation deviance curves for OS–LASSO–Cox and DFS–LASSO–Cox, respectively, under 10-fold cross-validation. Each colored curve corresponds to an individual regression coefficient in the LASSO–Cox model. A total of 11 coefficients were generated from seven baseline variables after dummy-variable encoding: CTS4 (3 coefficients), age (1), sex (1), TNM stage (2), tumor differentiation (2), tumor location (1), and postoperative chemotherapy (1). The *x*-axis represents log lambda, and the *y*-axis represents cross-validated deviance. The dashed vertical lines indicate the lambda at minimum deviance and the largest regularization parameter within one standard error of the minimum. Variables corresponding to the one-standard-error value were selected. (**C**,**D**) Coefficient path plots for OS–LASSO–Cox and DFS–LASSO–Cox. The *x*-axis represents log lambda, and the *y*-axis represents standardized coefficients. As lambda increases, coefficients are progressively shrunk toward zero. (**E**) Forest plot of the refitted LASSO–Cox models. Point estimates represent hazard ratios, horizontal lines indicate 95% confidence intervals, and the dashed vertical line corresponds to a hazard ratio of 1. (**F**) Nomogram derived from the exploratory OS–LASSO–Cox model. The nomogram is retained for descriptive completeness only and is not proposed for clinical use because the underlying full-trajectory exposure is time dependent.

**Figure 5:**
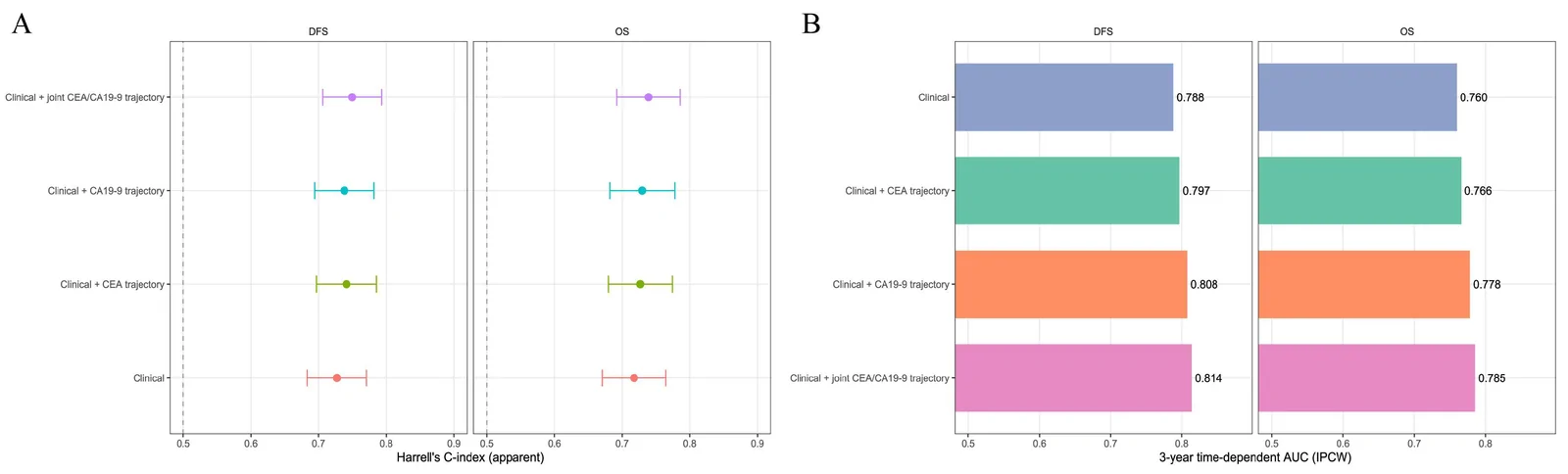
(**A**) Harrell’s C-index with 95% confidence intervals for the four Cox models, shown separately for overall survival (OS, **left**) and disease-free survival (DFS, **right**). (**B**) Three-year time-dependent AUC estimated by inverse probability of censoring weighting (IPCW) for the four Cox models. These full-cohort apparent performance displays are exploratory and should not be interpreted as out-of-fold validation.

**Figure 6:**
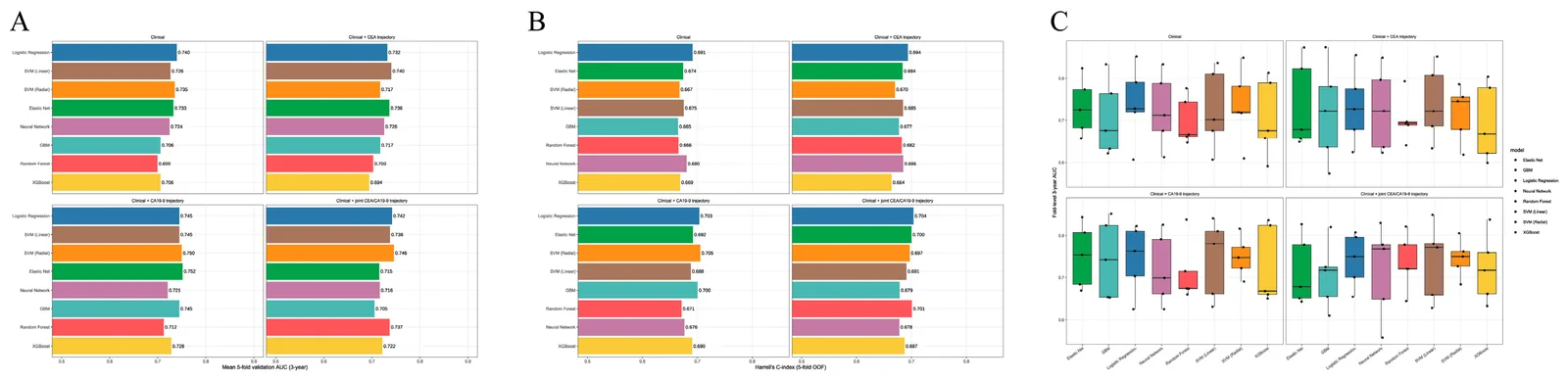
(**A**) Mean 3-year AUC across fivefold cross-validation for the eight machine learning algorithms under the four nested model combinations. (**B**) Out-of-fold (OOF) C-index for the eight machine learning algorithms under the four nested model combinations. (**C**) Variability of the fivefold AUC for each algorithm under the four nested model combinations. Dots represent individual fold results, and boxes indicate the distribution across folds. This panel belongs to the original full-trajectory exploratory workflow and is retained for transparency; corrected leakage-controlled performance is reported in Table 8.

**Figure 7:**
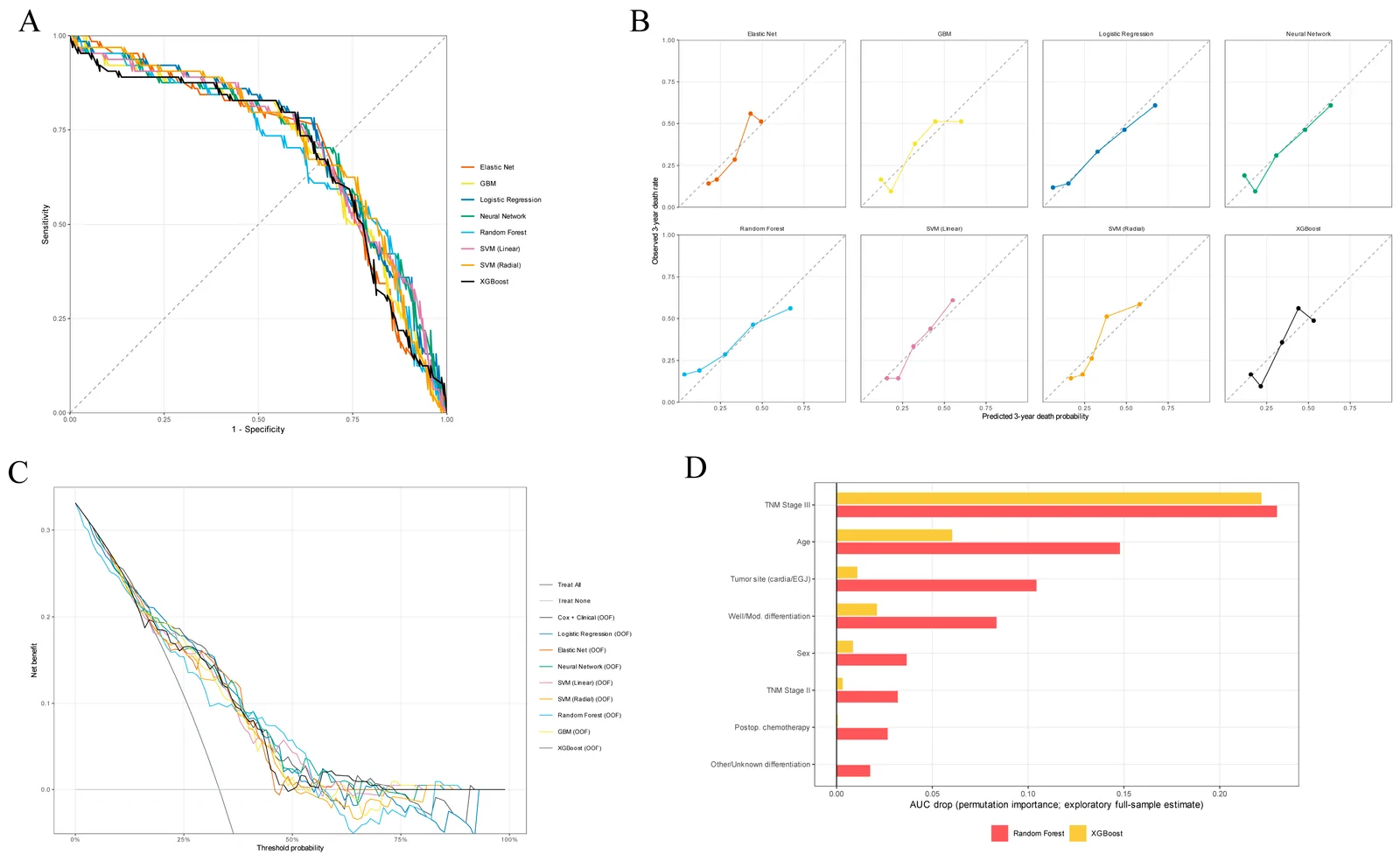
Models incorporating clinical variables: (**A**) ROC curves for 3-year mortality prediction (pROC, aggregated out-of-fold predictions). Each curve is constructed from out-of-fold predicted probabilities. The gray dashed line indicates the reference line for no discriminative ability (AUC = 0.5). (**B**) Calibration curves for the predicted 3-year mortality probability from the machine learning models. The *x*-axis shows predicted probability, and the *y*-axis shows the observed mortality rate in each group. The dashed diagonal line indicates perfect calibration; curves closer to this line reflect better agreement between predicted probabilities and actual risk. The gray dashed line indicates the ideal calibration line, where the predicted probability equals the observed event rate. (**C**) Decision curve analysis for 3-year mortality prediction. The *x*-axis represents threshold probability, and the *y*-axis represents net benefit. Model curves lying above the treat-all and treat-none strategies suggest potential clinical decision value within the corresponding threshold range. (**D**) Permutation feature importance for random forest and XGBoost. The *x*-axis represents the decrease in AUC after permuting each individual feature. This panel belongs to the original full-trajectory exploratory workflow and is retained for transparency; corrected leakage-controlled performance is reported in Table 8.

**Figure 8:**
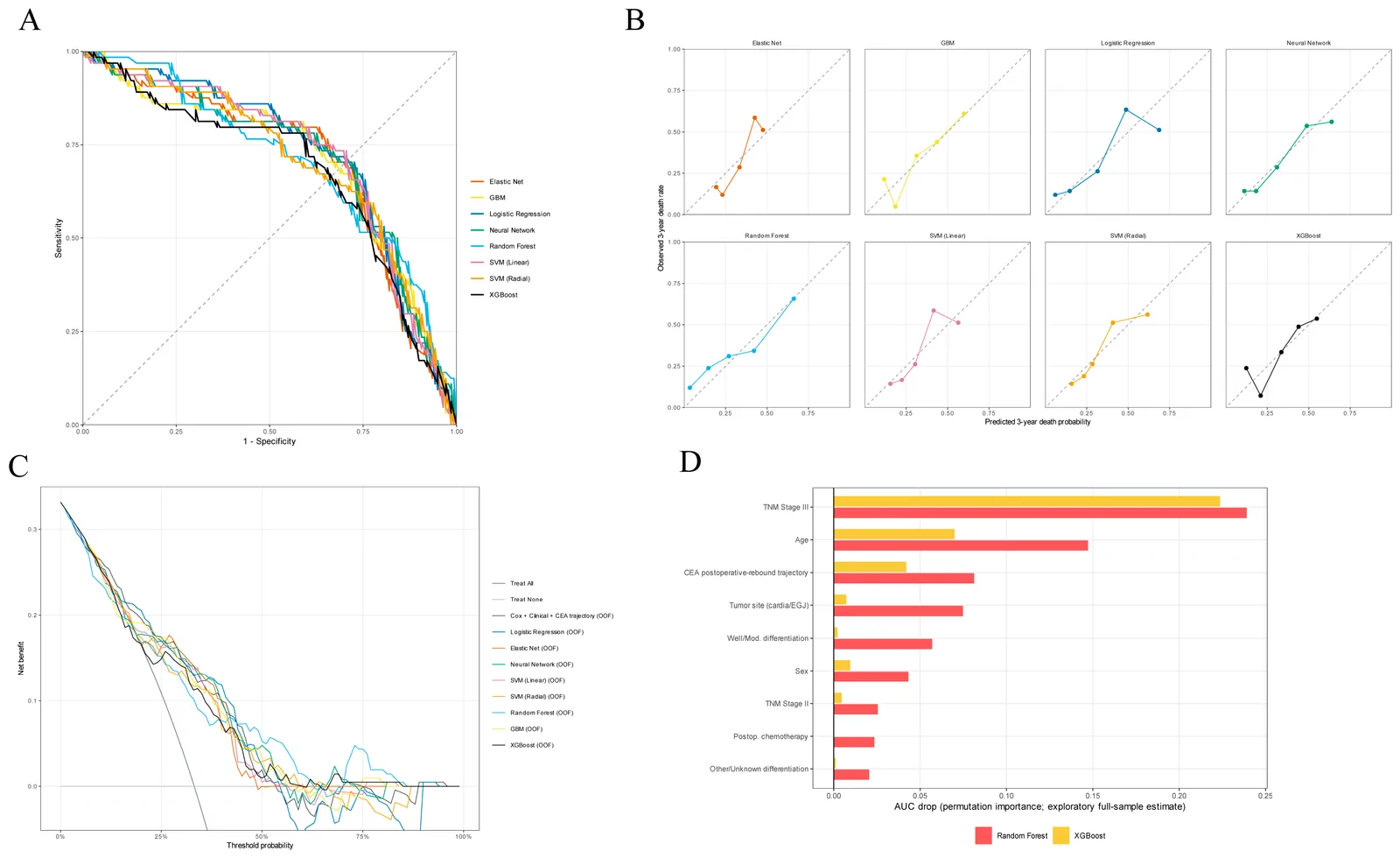
Models incorporating clinical variables plus the CEA trajectory only: (**A**) ROC curves for 3-year mortality prediction (pROC, aggregated out-of-fold predictions). Each curve is constructed from out-of-fold predicted probabilities. The gray dashed line indicates the reference line for no discriminative ability (AUC = 0.5). (**B**) Calibration curves for the predicted 3-year mortality probability from the machine learning models. The *x*-axis shows predicted probability, and the *y*-axis shows the observed mortality rate in each group. The dashed diagonal line indicates perfect calibration; curves closer to this line reflect better agreement between predicted probabilities and actual risk. The gray dashed line indicates the ideal calibration line, where the predicted probability equals the observed event rate. (**C**) Decision curve analysis for 3-year mortality prediction. The *x*-axis represents threshold probability, and the *y*-axis represents net benefit. Model curves lying above the treat-all and treat-none strategies suggest potential clinical decision value within the corresponding threshold range. (**D**) Permutation feature importance for random forest and XGBoost. The *x*-axis represents the decrease in AUC after permuting each individual feature. This panel belongs to the original full-trajectory exploratory workflow and is retained for transparency; corrected leakage-controlled performance is reported in Table 8.

**Figure 9:**
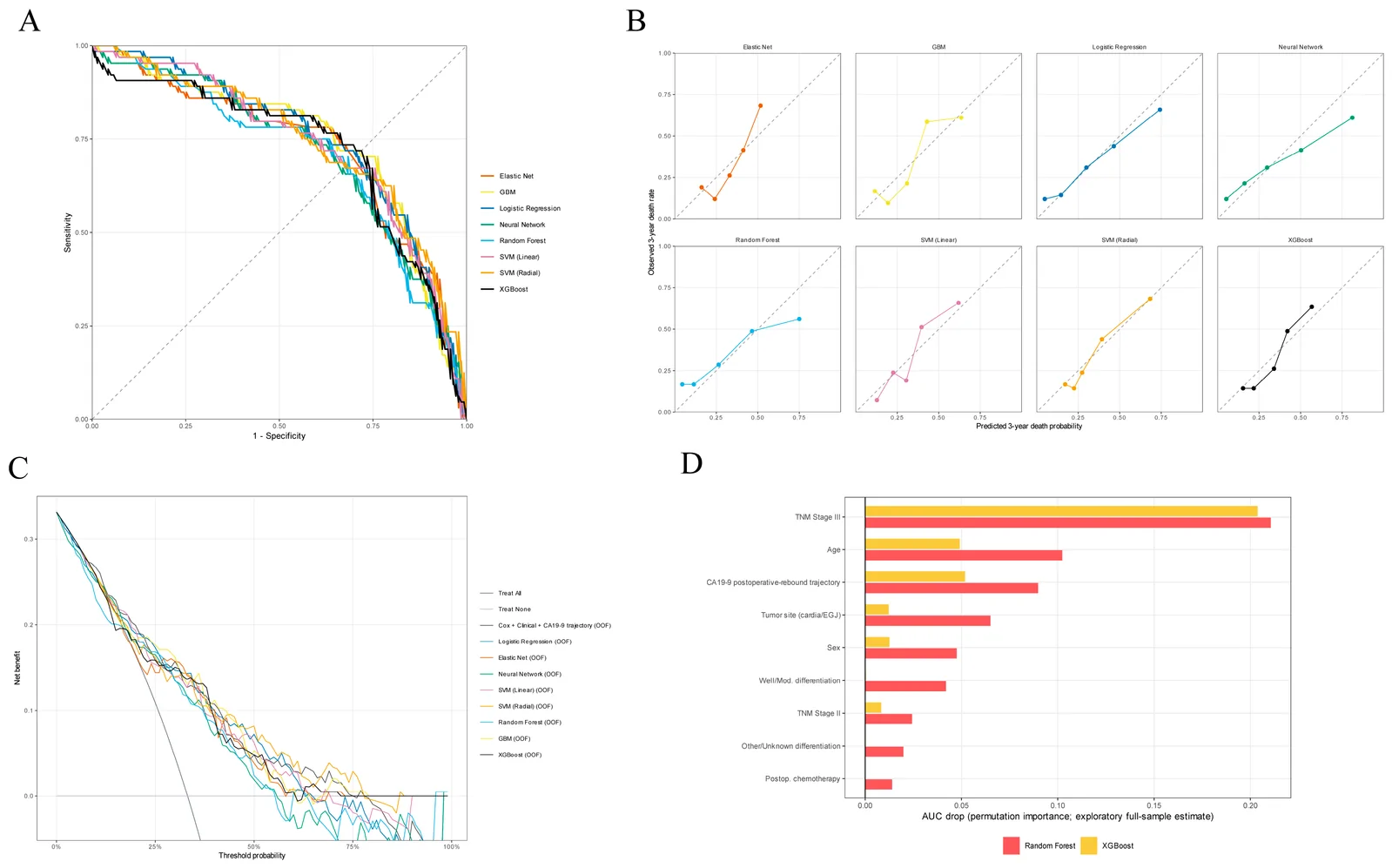
Models incorporating clinical variables plus the CA19-9 trajectory only: (**A**) ROC curves for 3-year mortality prediction (pROC, aggregated out-of-fold predictions). Each curve is constructed from out-of-fold predicted probabilities. The gray dashed line indicates the reference line for no discriminative ability (AUC = 0.5). (**B**) Calibration curves for the predicted 3-year mortality probability from the machine learning models. The *x*-axis shows predicted probability, and the *y*-axis shows the observed mortality rate in each group. The dashed diagonal line indicates perfect calibration; curves closer to this line reflect better agreement between predicted probabilities and actual risk. The gray dashed line indicates the ideal calibration line, where the predicted probability equals the observed event rate. (**C**) Decision curve analysis for 3-year mortality prediction. The *x*-axis represents threshold probability, and the *y*-axis represents net benefit. Model curves lying above the treat-all and treat-none strategies suggest potential clinical decision value within the corresponding threshold range. (**D**) Permutation feature importance for random forest and XGBoost. The *x*-axis represents the decrease in AUC after permuting each individual feature. This panel belongs to the original full-trajectory exploratory workflow and is retained for transparency; corrected leakage-controlled performance is reported in Table 8.

**Figure 10:**
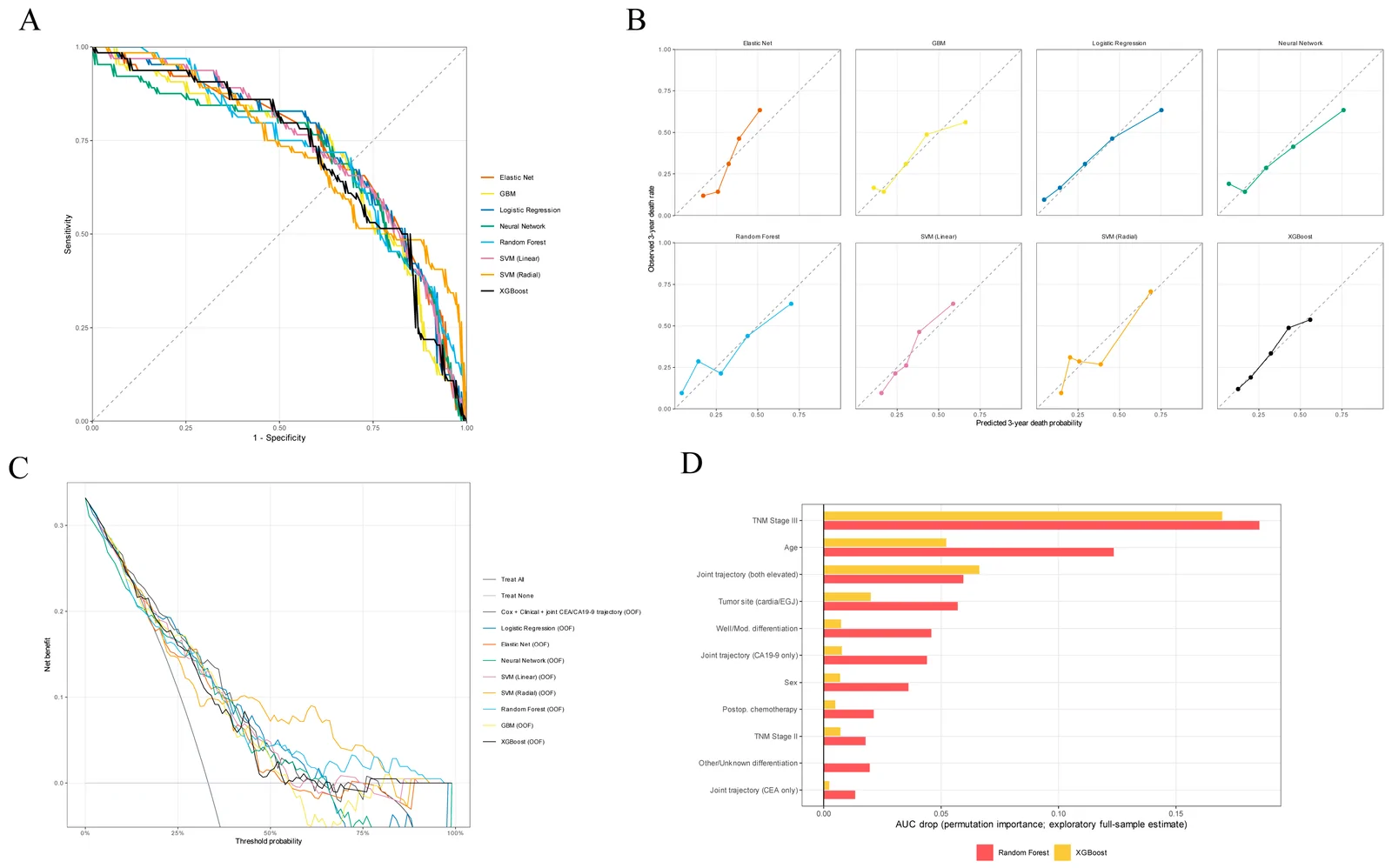
Models incorporating clinical variables plus the combined CEA/CA19-9 (CTS4) trajectory: (**A**) ROC curves for 3-year mortality prediction (pROC, aggregated out-of-fold predictions). Each curve is constructed from out-of-fold predicted probabilities. The gray dashed line indicates the reference line for no discriminative ability (AUC = 0.5). (**B**) Calibration curves for the predicted 3-year mortality probability from the machine learning models. The *x*-axis shows predicted probability, and the *y*-axis shows the observed mortality rate in each group. The dashed diagonal line indicates perfect calibration; curves closer to this line reflect better agreement between predicted probabilities and actual risk. The gray dashed line indicates the ideal calibration line, where the predicted probability equals the observed event rate. (**C**) Decision curve analysis for 3-year mortality prediction. The *x*-axis represents threshold probability, and the *y*-axis represents net benefit. Model curves lying above the treat-all and treat-none strategies suggest potential clinical decision value within the corresponding threshold range. (**D**) Permutation feature importance for random forest and XGBoost. The *x*-axis represents the decrease in AUC after permuting each individual feature. This panel belongs to the original full-trajectory exploratory workflow and is retained for transparency; corrected leakage-controlled performance is reported in Table 8.

**Figure 11:**
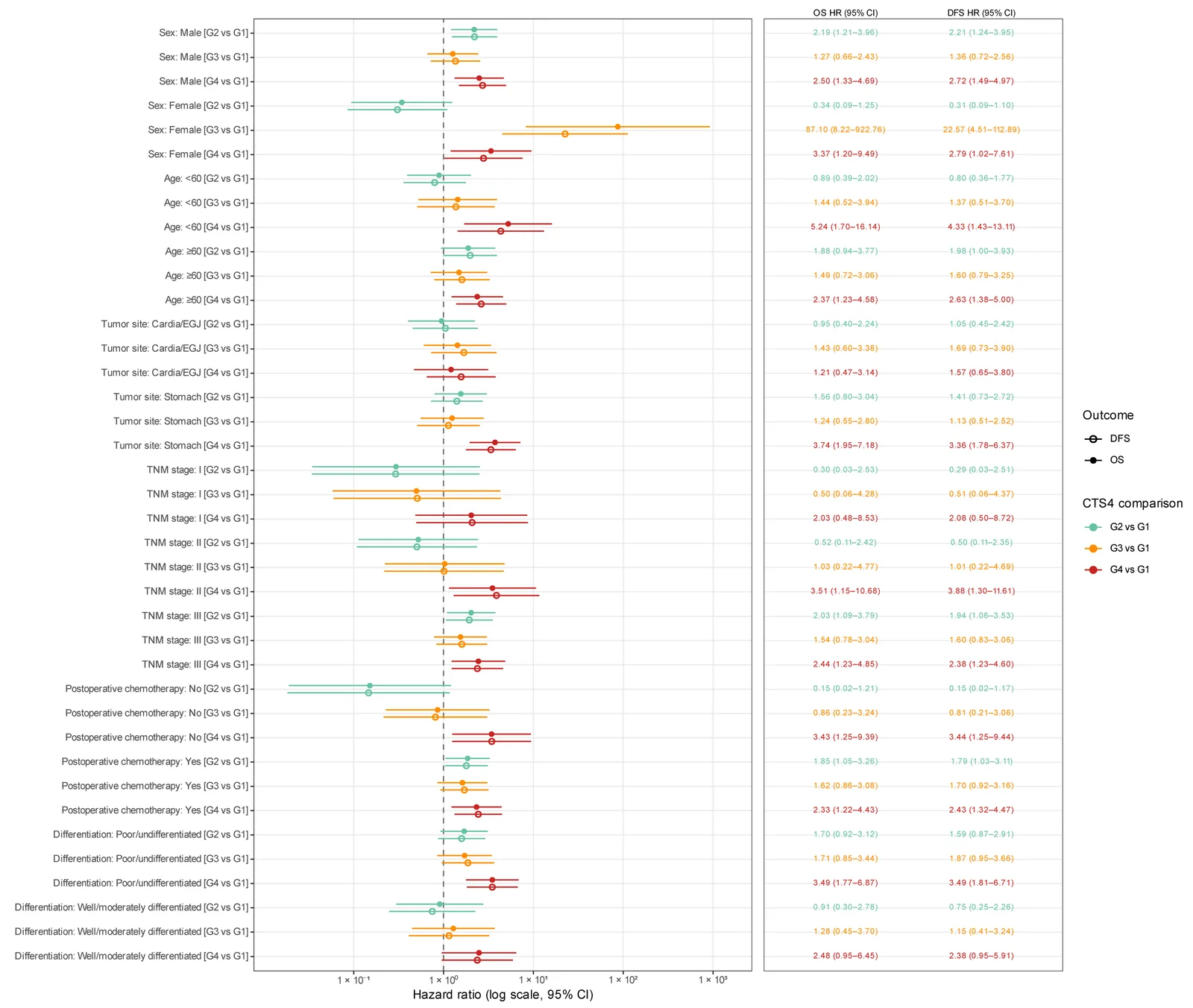
Subgroup forest plot for high-risk CTS4 G4 versus low-risk CTS4 G1. Point estimates represent hazard ratios, and horizontal lines indicate 95% confidence intervals. This panel was used to assess the direction and consistency of the exploratory G4 versus G1 association across clinical subgroups.

**Table 1:** Patients’ baseline characteristics of different trajectory subtypes.

Variables ^a^		Overall (*N* = 208)	G1 (Both Low-Stable) (*n* = 84)	G2 (CA19-9-Only Postoperative-Rebound) (*n* = 53)	G3 (CEA-Only Postoperative-Rebound) (*n* = 36)	G4 (Dual Postoperative-Rebound) (*n* = 35)	*p*-Values
Age		65.0 [57.0, 71.0]	62.0 [54.8, 69.0]	64.0 [56.0, 69.0]	68.0 [58.8, 73.0]	70.0 [64.0, 74.0]	0.002
Sex	Male	155 (74.5)	60 (71.4)	35 (66.0)	33 (91.7)	27 (77.1)	0.043
	Female	53 (25.5)	24 (28.6)	18 (34.0)	3 (8.3)	8 (22.9)	
TNM stage	I	59 (28.4)	25 (29.8)	16 (30.2)	9 (25.0)	9 (25.7)	0.267
	II	45 (21.6)	25 (29.8)	9 (17.0)	5 (13.9)	6 (17.1)	
	III	104 (50.0)	34 (40.5)	28 (52.8)	22 (61.1)	20 (57.1)	
T stage	1	43 (20.7)	21 (25.0)	12 (22.6)	6 (16.7)	4 (11.4)	0.305
	2	30 (14.4)	13 (15.5)	4 (7.5)	5 (13.9)	8 (22.9)	
	3	35 (16.8)	14 (16.7)	10 (18.9)	3 (8.3)	8 (22.9)	
	4	100 (48.1)	36 (42.9)	27 (50.9)	22 (61.1)	15 (42.9)	
N stage	0	84 (40.4)	37 (44.0)	24 (45.3)	11 (30.6)	12 (34.3)	0.079
	1	42 (20.2)	22 (26.2)	6 (11.3)	11 (30.6)	3 (8.6)	
	2	38 (18.3)	13 (15.5)	10 (18.9)	7 (19.4)	8 (22.9)	
	3	44 (21.2)	12 (14.3)	13 (24.5)	7 (19.4)	12 (34.3)	
Differentiation	Low-grade/undifferentiated or special types	129 (62.0)	55 (65.5)	34 (64.2)	21 (58.3)	19 (54.3)	0.653
	High/medium differentiation	73 (35.1)	25 (29.8)	18 (34.0)	15 (41.7)	15 (42.9)	
	Other/unspecified	6 (2.9)	4 (4.8)	1 (1.9)	0 (0.0)	1 (2.9)	
Tumor site	Esophagogastric junction/cardia	74 (35.6)	22 (26.2)	23 (43.4)	16 (44.4)	13 (37.1)	0.116
	Stomach	134 (64.4)	62 (73.8)	30 (56.6)	20 (55.6)	22 (62.9)	
Surgery type	Proximal gastrectomy	23 (11.1)	9 (10.7)	9 (17.0)	1 (2.8)	4 (11.4)	0.055
	Other/unspecified	2 (1.0)	0 (0.0)	1 (1.9)	1 (2.8)	0 (0.0)	
	Total gastrectomy	95 (45.7)	30 (35.7)	25 (47.2)	21 (58.3)	19 (54.3)	
	Distal gastrectomy	88 (42.3)	45 (53.6)	18 (34.0)	13 (36.1)	12 (34.3)	
Postoperative chemotherapy	No	41 (19.7)	15 (17.9)	10 (18.9)	6 (16.7)	10 (28.6)	0.541
	Yes	167 (80.3)	69 (82.1)	43 (81.1)	30 (83.3)	25 (71.4)	
Preoperative CEA		1.4 [0.8, 2.3]	1.0 [0.5, 1.4]	1.2 [0.8, 1.6]	2.8 [2.0, 8.2]	2.6 [1.9, 4.6]	<0.001
Preoperative CA19-9		8.1 [3.9, 18.3]	5.0 [2.6, 7.7]	17.8 [11.4, 33.8]	3.6 [2.0, 6.8]	28.5 [16.9, 101.9]	<0.001

^a^ Continuous variables were described as medians [Q1, Q3], and categorical variables as frequencies (%). Between-group comparisons were performed using the Kruskal–Wallis test for continuous variables and the chi-square test or Fisher’s exact test for categorical variables.

**Table 2:** Univariate Cox regression results for OS and DFS.

Variables ^a^	HR (95% CI) for OS	*p*-Value for OS	HR (95% CI) for DFS	*p*-Value for DFS
CTS4 G2, CA19-9 only rebound, vs. G1, both low-stable	1.34 (0.79–2.27)	0.271	1.30 (0.78–2.18)	0.310
CTS4 G3, CEA only rebound, vs. G1	1.44 (0.81–2.56)	0.210	1.49 (0.85–2.61)	0.162
CTS4 G4, dual rebound, vs. G1	2.58 (1.51–4.41)	<0.001	2.66 (1.59–4.46)	<0.001
Age	1.02 (1.00–1.04)	0.040	1.02 (1.00–1.04)	0.033
Sex (female vs. male)	0.84 (0.52–1.35)	0.468	0.82 (0.52–1.30)	0.404
TNM stage II vs. I	2.64 (1.22–5.72)	0.014	2.83 (1.32–6.10)	0.008
TNM stage III vs. I	6.27 (3.23–12.19)	<0.001	7.27 (3.75–14.09)	<0.001
T stage (For every level up)	1.66 (1.36–2.02)	<0.001	1.71 (1.40–2.08)	<0.001
N stage (For every level up)	1.72 (1.45–2.03)	<0.001	1.81 (1.54–2.13)	<0.001
Differentiation (high/moderate vs. low/undifferentiated)	0.61 (0.40–0.95)	0.030	0.63 (0.41–0.97)	0.036
Differentiation (other vs. low/undifferentiated)	0.60 (0.15–2.43)	0.471	0.91 (0.29–2.88)	0.869
Tumor location (stomach vs. cardia/GEJ)	0.84 (0.56–1.26)	0.398	0.76 (0.51–1.12)	0.168
Surgery type (other vs. proximal gastrectomy)	1.44 (0.18–11.73)	0.732	1.97 (0.24–15.97)	0.526
Surgery type (total gastrectomy vs. proximal gastrectomy)	2.31 (1.05–5.08)	0.037	2.14 (1.02–4.49)	0.044
Surgery type (distal gastrectomy vs. proximal gastrectomy)	1.48 (0.66–3.34)	0.339	1.37 (0.64–2.93)	0.421
Postoperative chemotherapy (yes vs. no)	1.04 (0.63–1.70)	0.882	1.15 (0.70–1.87)	0.585
Preoperative CEA (log)	1.31 (1.04–1.65)	0.021	1.38 (1.09–1.74)	0.007
Preoperative CA19-9 (log)	1.26 (1.08–1.48)	0.004	1.24 (1.06–1.45)	0.006

^a^ HR: Hazard Ratio; CI: Confidence Interval.

**Table 3:** Results of the LASSO–Cox multivariate regression analysis.

Variables ^a^	HR (95% CI) for OS	HR (95% CI) for DFS	*p*-Value for OS	*p*-Value for DFS
CTS4 G2 (CA19-9-only postoperative-rebound group) vs. G1 (both low-stable)	1.36 (0.80–2.32)	1.33 (0.79–2.24)	0.257	0.279
CTS4 G3, CEA only rebound, vs. G1	1.18 (0.65–2.14)	1.21 (0.67–2.18)	0.594	0.522
CTS4 G4, dual rebound, vs. G1	2.25 (1.26–4.04)	2.29 (1.30–4.02)	0.006	0.004
Age	1.01 (0.99–1.04)	1.01 (0.99–1.04)	0.249	0.235
TNM stage II vs. I	2.67 (1.22–5.84)	2.85 (1.31–6.18)	0.014	0.008
TNM stage III vs. I	6.31 (3.24–12.28)	7.26 (3.74–14.10)	<0.001	<0.001

^a^ λ was determined by 10-fold cross-validation (1-SE criterion). HR, hazard ratio; CI, confidence interval. Selected variables: CTS4, age, and TNM stage (G1 double-low as reference).

**Table 4:** Standard multivariable Cox regression analysis of CEA trajectory and survival outcomes.

Variables	HR (95% CI) for OS	*p*-Value for OS	HR (95% CI) for DFS	*p*-Value for DFS
Age	1.02 (1.00–1.04)	0.086	1.02 (0.99–1.04)	0.157
Sex (female vs. male)	1.14 (0.70–1.87)	0.595	1.21 (0.75–1.95)	0.445
TNM stage II vs. I	2.86 (1.28–6.43)	0.011	3.35 (1.49–7.53)	0.003
TNM stage III vs. I	6.61 (3.27–13.37)	<0.001	8.20 (4.01–16.74)	<0.001
Differentiation (other vs. low/undifferentiated)	1.07 (0.25–4.59)	0.932	2.23 (0.66–7.60)	0.198
Differentiation (high/moderate vs. low/undifferentiated)	0.67 (0.42–1.08)	0.103	0.73 (0.46–1.17)	0.188
Tumor location (stomach vs. cardia/GEJ)	1.08 (0.70–1.66)	0.730	0.98 (0.64–1.48)	0.906
Postoperative chemotherapy (yes vs. no)	0.63 (0.37–1.07)	0.087	0.65 (0.39–1.10)	0.109
CEA postoperative rebound vs. low-stable	1.41 (0.91–2.20)	0.128	1.51 (0.98–2.33)	0.063

**Table 5:** Standard multivariable Cox regression analysis of CA19-9 trajectory and survival outcomes.

Variables	HR (95% CI) for OS	*p*-Value for OS	HR (95% CI) for DFS	*p*-Value for DFS
Age	1.02 (1.00–1.05)	0.043	1.02 (1.00–1.04)	0.051
Sex (female vs. male)	0.94 (0.57–1.57)	0.832	1.02 (0.62–1.67)	0.942
TNM stage II vs. I	2.95 (1.30–6.68)	0.010	3.28 (1.45–7.41)	0.004
TNM stage III vs. I	6.93 (3.41–14.09)	<0.001	8.28 (4.06–16.87)	<0.001
Differentiation (other vs. low/undifferentiated)	0.79 (0.19–3.39)	0.755	1.77 (0.53–5.94)	0.354
Differentiation (high/moderate vs. low/undifferentiated)	0.64 (0.40–1.03)	0.068	0.70 (0.44–1.11)	0.127
Tumor location (stomach vs. cardia/GEJ)	1.18 (0.76–1.83)	0.465	1.05 (0.69–1.61)	0.814
Postoperative chemotherapy (yes vs. no)	0.61 (0.36–1.04)	0.071	0.64 (0.38–1.08)	0.095
CA19-9 postoperative rebound vs. low-stable	1.68 (1.09–2.60)	0.019	1.58 (1.04–2.40)	0.033

**Table 6:** Standard multivariable Cox regression analysis of combined CEA/CA19-9 trajectory subtypes (CTS4) and survival outcomes.

Variables	HR (95% CI) for OS	*p*-Value for OS	HR (95% CI) for DFS	*p*-Value for DFS
CTS4 G2, CA19-9 only elevated, vs. G1	1.53 (0.88–2.69)	0.133	1.40 (0.81–2.41)	0.228
CTS4 G3, CEA only elevated, vs. G1	1.26 (0.68–2.31)	0.461	1.31 (0.72–2.38)	0.383
CTS4 G4, both elevated, vs. G1	2.35 (1.28–4.30)	0.006	2.35 (1.31–4.20)	0.004
Age	1.02 (0.99–1.04)	0.166	1.01 (0.99–1.04)	0.247
Sex (female vs. male)	0.96 (0.58–1.60)	0.874	1.03 (0.63–1.70)	0.894
TNM stage II vs. I	3.07 (1.35–6.94)	0.007	3.51 (1.55–7.93)	0.003
TNM stage III vs. I	6.82 (3.36–13.86)	<0.001	8.34 (4.07–17.07)	<0.001
Differentiation (high/moderate vs. low/undifferentiated)	0.66 (0.41–1.07)	0.093	0.73 (0.45–1.18)	0.198
Differentiation (other vs. low/undifferentiated)	0.95 (0.22–4.13)	0.944	2.10 (0.61–7.19)	0.236
Tumor location (stomach vs. cardia/GEJ)	1.18 (0.76–1.83)	0.470	1.05 (0.69–1.61)	0.818
Postoperative chemotherapy (yes vs. no)	0.62 (0.36–1.05)	0.076	0.64 (0.38–1.08)	0.092

**Table 7:** Comparison of apparent C-indices among the nested exploratory models, *N* = 208.

Outcome ^a^	Model	Concordance Index (95% CI)	3-Year AUC
Overall survival (OS)	Clinical variables	0.718 (0.671–0.764)	0.760
Overall survival (OS)	Clinical variables + only CEA trajectory	0.727 (0.680–0.774)	0.766
Overall survival (OS)	Clinical variables + only CA19-9 trajectory	0.730 (0.682–0.778)	0.778
Overall survival (OS)	Clinical variables + combined CEA/CA19-9 (CTS4) trajectory	0.739 (0.692–0.786)	0.785
Disease-free survival (DFS)	Clinical variables	0.727 (0.683–0.771)	0.788
Disease-free survival (DFS)	Clinical variables + only CEA trajectory	0.741 (0.697–0.786)	0.797
Disease-free survival (DFS)	Clinical variables + only CA19-9 trajectory	0.738 (0.695–0.782)	0.808
Disease-free survival (DFS)	Clinical variables + combined CEA/CA19-9 (CTS4) trajectory	0.750 (0.706–0.793)	0.814

^a^ C-index, Harrell’s concordance index, with 95% confidence intervals estimated by bootstrap resampling. Clinical variables included age, sex, TNM stage, differentiation grade, tumor location, and postoperative chemotherapy.

**Table 8:** Leakage-controlled out-of-fold performance in the 12-month landmark cohort.

Model	Input/Framework	OOF C-Index	AUC at 24 Months After Landmark
Cox clinical + baseline	Clinical plus baseline markers	0.574	0.601
Cox clinical + baseline + CTS4	Clinical plus baseline markers plus fold-specific CTS4	0.589	0.635
Logistic Regression	Clinical plus baseline markers plus fold-specific CTS4	0.614	0.643
Elastic Net	Clinical plus baseline markers plus fold-specific CTS4	0.612	0.642
SVM Linear	Clinical plus baseline markers plus fold-specific CTS4	0.505	0.484
SVM Radial	Clinical plus baseline markers plus fold-specific CTS4	0.606	0.602
Random Forest	Clinical plus baseline markers plus fold-specific CTS4	0.629	0.640
GBM	Clinical plus baseline markers plus fold-specific CTS4	0.596	0.611
Neural Network	Clinical plus baseline markers plus fold-specific CTS4	0.500	0.501
XGBoost	Clinical plus baseline markers plus fold-specific CTS4	0.623	0.648

OOF denotes out-of-fold prediction. The C-index was calculated from held-out predictions against observed overall survival. The AUC endpoint was death within 24 months after the 12-month landmark, equivalent to 36 months after surgery.

**Table 9:** Subgroup analysis results for Group 4 (both elevated) versus Group 1 (both low-stable) in overall survival.

Outcome ^a^	Subgroup	Stratum	N	Events	HR (95% CI)	*p*-Value Within Subgroup	Interaction *p*-Value
OS	Sex	male	155	76	2.50 (1.33–4.69)	0.004	<0.001
		female	53	22	3.37 (1.20–9.49)	0.021	
	Age	<60	81	34	5.24 (1.70–16.14)	0.004	0.193
		≥60	127	64	2.37 (1.23–4.58)	0.010	
	Tumor site	Cardia/gastroesophageal junction	74	38	1.21 (0.47–3.14)	0.691	0.025
		Gastric body/pylorus	134	60	3.74 (1.95–7.18)	<0.001	
	TNM stage	stage I	59	10	2.03 (0.48–8.53)	0.332	0.119
		stage II	45	18	3.51 (1.15–10.68)	0.027	
		stage III	104	70	2.44 (1.23–4.85)	0.011	
	Postoperative chemotherapy	No chemotherapy	41	20	3.43 (1.25–9.39)	0.017	0.048
		Chemotherapy	167	78	2.33 (1.22–4.43)	0.010	
	Differentiation	Low-grade/undifferentiated or special types	129	68	3.49 (1.77–6.87)	<0.001	0.009
		High/medium differentiation	73	28	2.48 (0.95–6.45)	0.063	
		Other/unspecified	6	2	—		
DFS	Sex	male	155	81	2.72 (1.49–4.97)	0.001	<0.001
		female	53	23	2.79 (1.02–7.61)	0.046	
	Age	<60	81	36	4.33 (1.43–13.11)	0.009	0.113
		≥60	127	68	2.63 (1.38–5.00)	0.003	
	Tumor site	Cardia/gastroesophageal junction	74	42	1.57 (0.65–3.80)	0.316	0.047
		Gastric body/pylorus	134	62	3.36 (1.78–6.37)	<0.001	
	TNM stage	stage I	59	10	2.08 (0.50–8.72)	0.316	0.120
		stage II	45	19	3.88 (1.30–11.61)	0.015	
		stage III	104	75	2.38 (1.23–4.60)	0.010	
	Postoperative chemotherapy	No chemotherapy	41	20	3.44 (1.25–9.44)	0.017	0.031
		Chemotherapy	167	84	2.43 (1.32–4.47)	0.004	
	Differentiation	Low-grade/undifferentiated or special types	129	71	3.49 (1.81–6.71)	<0.001	0.014
		High/medium differentiation	73	30	2.38 (0.95–5.91)	0.063	
		Other/unspecified	6	3	—		

^a^ Cox Subgroup Analysis: Within each subgroup, Surv(os_months, death_status) ~ CTS4, with G1 as the reference group; Interaction Test: Likelihood ratio test (LRT), adjusting for clinical covariates other than the subgroup variable itself.

**Table 10:** Sensitivity analysis using multiple imputation and the fully adjusted exploratory CTS4 Cox models for overall survival and disease-free survival.

Variables	HR (95% CI) for OS	*p*-Value for OS	HR (95% CI) for DFS	*p*-Value for DFS
CTS4 G2, CA19-9 only rebound, vs. G1, both low-stable	1.53 (0.87–2.71)	0.137	1.4 (0.80–2.43)	0.231
CTS4 G3, CEA only rebound, vs. G1	1.26 (0.68–2.33)	0.463	1.31 (0.71–2.39)	0.385
CTS4 G4, dual rebound, vs. G1	2.35 (1.27–4.34)	0.007	2.35 (1.30–4.23)	0.005
Age	1.02 (0.99–1.04)	0.17	1.01 (0.99–1.04)	0.25
Sex (female vs. male)	0.96 (0.57–1.61)	0.874	1.03 (0.63–1.71)	0.894
TNM stage II vs. I	3.07 (1.34–7.02)	0.009	3.51 (1.53–8.02)	0.003
TNM stage III vs. I	6.82 (3.32–14.01)	<0.001	8.34 (4.04–17.24)	<0.001
Differentiation (high/moderate vs. low/undifferentiated)	0.66 (0.40–1.08)	0.097	0.73 (0.45–1.18)	0.201
Differentiation (other vs. low/undifferentiated)	0.95 (0.21–4.22)	0.944	2.1 (0.60–7.32)	0.239
Tumor location (stomach vs. cardia/GEJ)	1.18 (0.75–1.84)	0.472	1.05 (0.68–1.62)	0.818
Postoperative chemotherapy (yes vs. no)	0.62 (0.36–1.06)	0.079	0.64 (0.37–1.08)	0.095

## Data Availability

Due to ethical restrictions, the data that support the findings of this study are not publicly available. However, they can be obtained from the corresponding author, Tong Zhou, upon reasonable request.

## Supplementary Materials

The following supporting information can be downloaded at: https://www.mdpi.com/article/10.3390/biomedicines14102175/s1, Table S1. Available-data comparison between the 208 analyzed patients and 99 nonincluded patients with baseline information retained in the analytic extraction; Table S2. Missing longitudinal CEA and CA19-9 values and their prespecified handling in the 208-patient trajectory cohort; Table S3. Twelve-month landmark Cox analysis and incremental predictive performance.

## Abbreviations

CEA	Carcinoembryonic Antigen
CA19-9	Carbohydrate Antigen 19-9
CA72-4	Carbohydrate Antigen 72-4
DFS	Disease-Free Survival
OS	Overall Survival
HR	Hazard Ratio
ng/mL	Nanograms Per Milliliter
ARI	Adjusted Rand Indices
OOF	Out-of-Fold Predictions
IPCW	Inverse Probability of Censoring Weighting
PET-CT	Positron Emission Tomography-Computed Tomography
NCCN	National Comprehensive Cancer Network
AJCC	American Joint Committee on Cancer
