# Supplementary material for: Joint Dynamic Trajectories of CEA and CA19-9 After Gastric Cancer Surgery: Prognostic Associations and a 12-Month Landmark Analysis
Source: Biomedicines. 2026 Sep 25;14(10):2175. doi: 10.3390/biomedicines14102175 (PMC13701777; doi:10.3390/biomedicines14102175)
Supplement: Supplementary file 1 [file biomedicines-14-02175-s001.zip › biomedicines-4564381-supplementary.pdf]

Table S1. Available-data comparison between the 208 analyzed patients and 99 nonincluded patients with baseline information retained in the analytic extraction.

Characteristic	Analyzed cohort, n = 208	Nonincluded available subset, n = 99	p value
Age, years	65.0 [57.0, 71.0]	66.0 [59.0, 71.5]	0.189
Male sex	155 [74.5%]	65 [65.7%]	0.140
TNM stage I / II / III	59 / 45 / 104	33 / 22 / 44	0.609
Low or undifferentiated / high or moderate / other	129 / 73 / 6	58 / 34 / 6	0.392
Stomach / cardia or gastroesophageal junction	134 / 74	56 / 43	0.230
Postoperative chemotherapy, yes	167 [80.3%]	70 of 98 [71.4%]	0.113
Observed paired marker time points	4 [3, 5]	3 [2, 4]	<0.001

Note: Individual-level baseline data were not retained for all 778 excluded patients. This table therefore describes only the 99 nonincluded patients available in the analytic extraction and is not a complete screened-cohort comparison. Survival was available for only 20 of these 99 patients and was not compared.

Table S2. Missing longitudinal CEA and CA19-9 values and their prespecified handling in the 208-patient trajectory cohort.

Marker	Time point	Missing, n [%]	Internal interpolation, n	Remaining imputation, n
CEA	T1	0 [0.0]	0	0
CEA	T2	45 [21.6]	35	10
CEA	T3	24 [11.5]	14	10
CEA	T4	42 [20.2]	13	29
CEA	T5	88 [42.3]	0	88
CA19-9	T1	0 [0.0]	0	0
CA19-9	T2	45 [21.6]	35	10
CA19-9	T3	24 [11.5]	14	10
CA19-9	T4	41 [19.7]	13	28
CA19-9	T5	88 [42.3]	0	88

Note: Internal interpolation was used only when the immediately preceding and following planned measurements were both observed. Remaining missing values were handled by the prespecified imputation strategy. Sixty patients required at least one interpolation and 98 required at least one imputed value.

Table S3. Twelve-month landmark Cox analysis and incremental predictive performance.

Outcome	Comparison	HR	95% CI	p value	Model performance
OS	G2 vs G1	1.58	0.70 to 3.56	0.272	
OS	G3 vs G1	1.00	0.38 to 2.64	0.996	
OS	G4 vs G1	1.81	0.67 to 4.89	0.242	C-index 0.689 to 0.710; AUC 0.736 to 0.760; LRT p = 0.583
DFS	G2 vs G1	1.45	0.65 to 3.22	0.364	
DFS	G3 vs G1	1.04	0.41 to 2.60	0.941	
DFS	G4 vs G1	1.90	0.74 to 4.86	0.183	C-index 0.711 to 0.723; AUC 0.755 to 0.769; LRT p = 0.569
Validation	Leakage-controlled Cox plus CTS4				OOF C-index 0.589; 24-month landmark AUC 0.635
